# The adhesion modulation protein, AmpA localizes to an endocytic compartment and influences substrate adhesion, actin polymerization and endocytosis in vegetative Dictyostelium cells

**DOI:** 10.1186/1471-2121-13-29

**Published:** 2012-11-05

**Authors:** Elizabeth F Noratel, Chere’ L Petty, Jessica S Kelsey, Hoa N Cost, Nisha Basappa, Daphne D Blumberg

**Affiliations:** 1 Department of Biological Sciences, University of Maryland, Baltimore County 1000 Hilltop Circle, Baltimore, MD, 21250, USA

**Keywords:** Actin polymerization, Endocytosis, Substrate adhesion, Migration, Dictyostelium discoideum

## Abstract

**Background:**

AmpA is a secreted 24Kd protein that has pleiotropic effects on *Dictyostelium* development. Null mutants delay development at the mound stage with cells adhering too tightly to the substrate. Prestalk cells initially specify as prespore cells and are delayed in their migration to the mound apex. Extracellular AmpA can rescue these defects, but AmpA is also necessary in a cell autonomous manner for anterior like cells (ALCs) to migrate to the upper cup. The ALCs are only 10% of the developing cell population making it difficult to study the cell autonomous effect of AmpA on the migration of these cells. AmpA is also expressed in growing cells, but, while it contains a hydrophobic leader sequence that is cleaved, it is not secreted from growing cells. This makes growing cells an attractive system for studying the cell autonomous function of AmpA.

**Results:**

In growing cells AmpA plays an environment dependent role in cell migration. Excess AmpA facilitates migration on soft, adhesive surfaces but hinders migration on less adhesive surfaces. AmpA also effects the level of actin polymerization. Knockout cells polymerize less actin while over expressing cells polymerize more actin than wild type. Overexpression of AmpA also causes an increase in endocytosis that is traced to repeated formation of multiple endocytic cups at the same site on the membrane. Immunofluorescence analysis shows that AmpA is found in the Golgi and colocalizes with calnexin and the slow endosomal recycling compartment marker, p25, in a perinuclear compartment. AmpA is found on the cell periphery and is endocytically recycled to the perinuclear compartment.

**Conclusion:**

AmpA is processed through the secretory pathway and traffics to the cell periphery where it is endocytosed and localizes to what has been defined as a slow endosomal recycling compartment. AmpA plays a role in actin polymerization and cell substrate adhesion. Additionally AmpA influences cell migration in an environment dependent manner. Wild type cells show very little variation in migration rates under the different conditions examined here, but either loss or over expression of AmpA cause significant substrate and environment dependent changes in migration.

## Background

Cell migration plays a vital role in many cellular processes, including neural crest migration and gastrulation in the embryo, immune responses and cancer metastasis. In order for these processes to proceed, there has to be an optimal level of cell adhesion [1]. If the adhesion to the substrate is too weak relative to the contractile force exerted by the cell, cells spread inefficiently and traction is reduced. Strong adhesion relative to contractile force causes the cells to spread correctly but to lose the ability to regulate the adhesion and the cells remain adhered to the substrate. Both of the above cases lead to inefficient motility. An optimal level of actin polymerization is also required [2].

Actin polymerization takes place at the leading edge of migrating cells [3]. This process is tightly controlled and involves severing proteins, capping proteins and nucleating proteins (reviewed in [4]). In mammalian cells, integrins bind to the extracellular matrix, sending signals from the matrix back into the cell. Depending on the signal from the ECM, the cell either will adhere to the matrix and continue growth, division or migration or will differentiate [5]. Cells form focal adhesions at sites of integrin binding which recruit actin binding proteins, such as Arp2/3, that induce actin polymerization [6]. Thus, actin polymerization and adhesion are irrevocably coupled in the process of cell migration.

The model organism *Dictyostelium discoideum* is uniquely suited for the study of cell migration and chemotaxis. It is a haploid protist which is ideal for genetic manipulation, and its genome has been sequenced [7]. Its life cycle consists of a vegetative state in which it survives in nature on the forest floor. It feeds by chemotacting to and consuming bacteria [8]. When resources become scarce, the program of development begins [9].

There are several points during development where the cells must migrate in order for development to proceed correctly. As nutrients become scarce, a progenitor cell secretes a signal indicating to other cells that starvation is imminent. Cells receiving this signal begin to secrete cAMP, a chemoattractant signal for aggregation. Cells then migrate into aggregation centers, initially moving as single cells, but later in the process they form end to end and side to side contacts, streaming in a “daisy chain” like manner to create the multi-cellular mounds (reviewed in [9,10]). At this point the cells differentiate into pre-spore and pre-stalk cells, along with a subset of pre-stalk cells called Anterior Like Cells (ALC’s). The cells migrate through the mound to their appropriate positions [11]. As development continues, the ALC’s prove to be the most migratory of the cells. They are initially found at the mound periphery and then a subset of the ALCs migrate to the tip of the mound. Their swirling migration pattern is a driving force in culmination, where they form the upper and lower cups supporting the sorus and the basal disk supporting the stalk [12,13].

The question that arises is how cells regulate their adhesions, both to the wide variety of substrates that *Dictyostelium* finds in the forest and to other cells during multicellular development. No true integrins have been found in the *Dictyostelium* genome, although some proteins with homology to integrin β have been discovered to have roles in cell adhesion [14,15]. *Dictyostelium* has genes coding for homologues of paxillin and talin proteins, suggestive of an ability to form focal adhesions, although evidence for the presence of focal adhesions is unclear [16,17]. Interestingly, there are two different talin genes. The talB gene functions primarily in development when cells are migrating over each other during morphogenesis; the talA gene functions primarily during growth when cells migrate on a wide variety of substrates from dirt to cellulous nitrate filters to glass and plastics [17]. Recent work seems to indicate that the two talin proteins also have some overlapping functions [18]. How cells modify their adhesions to accommodate so many different substrates is not trivial.

We have previously described a novel adhesion protein, Adhesion Modulation Protein A (AmpA), that plays a role in cell migration and adhesion during development [19-21]. During development, about 70% of the AmpA protein is secreted, but a small proportion remains cell associated [19]. When the *ampA* gene is knocked out, cells reach mound stage at the same time as wildtype, but there is a 4 to 6 hour delay in tip formation compared to wild type. Cells at the mound periphery that would normally differentiate as prestalk cells initially express prespore genes. They remain at the mound periphery and are delayed in their migration to the tip of the mound. A large percentage of the cells show significantly increased adhesion to the substrate in the AmpA knockouts suggesting that the increased cell substrate adhesion may be responsible for the delayed migration of the prestalk cells to the mound apex [19,20]. In chimeras of wild type and *ampA* null cells, most of these defects are rescued by the presence of secreted AmpA protein suggesting that AmpA acts extracellularly. However, AmpA also plays an internal, cell autonomous role in regulating the migration of ALCs. In wild type-AmpA null chimeras, ALCs carrying the *ampA* null mutation never migrate to the upper cup region. They remain at the base of the mound. The upper cup region forms entirely from wildtype cells in the chimeras. Using reporter constructs, it was shown that AmpA is expressed in all growing cells and in scattered cells during early development, but its expression becomes entirely localized to the ALCs as development proceeds [22]. Since the ALC’s comprise only about 10% of the cells in the final fruiting body, studying the role of AmpA in the migration of these cells is difficult [23].

During vegetative growth, AmpA is expressed in all cells especially as they reach high density and, unlike in developing cells, AmpA is not secreted during growth [19,22]. It contains a hydrophobic leader sequence which is cleaved but the AmpA protein is never found free in the media in growing cells. Thus, growing cells represent an opportunity to study the cell autonomous functions of AmpA. Here, we demonstrate a role for the presence of AmpA causing a decrease in cell adhesion to the substrate and thereby promoting cell migration. Depending on environmental conditions, an optimal amount of AmpA is required for chemotaxis of growing cells to folic acid which is thought to be the bacterial chemoattractant [24]. Loss of AmpA or excessive AmpA results in cells that are only able to migrate efficiently under certain conditions. We also show a role for AmpA in influencing actin polymerization and we show that excess AmpA can increase the amount of endocytosis. Based on the localization of AmpA on the cell surface and its endocytosis and subsequent localization to a perinuclear recycling compartment, we postulate a role for AmpA in possibly controlling the recycling of an adhesion receptor or acting as a signaling molecule.

## Results

### The *ampA* gene influences the migration of growing cells to folic acid

In nature *Dictyostelium* lives on the forest floor and feeds on bacteria which it locates by chemotaxis to folic acid present in bacteria. In the laboratory *Dictyostelium* amoebae can be grown on agar plates in association with bacteria. Under these conditions, single *Dictyostelium* cells grow by ingesting the bacteria by phagocytosis, clearing plaques in the bacterial lawn as cells migrate out radially to forage for more bacteria. The size of the plaques can reflect the rate of phagocytosis, the growth rate of the cells or the rate of migration of the cells out into the bacterial lawn. We have previously shown that plaques formed by *ampA* null cells are significantly smaller than those formed by wild type cells [25] and summarized in Table 1. By contrast the plaques formed by the AmpA overexpressing strain are significantly larger than the wild type plaques [25] and summarized in Table 1. The rate of phagocytosis as measured by the uptake of latex beads by *ampA* null and overexpressing cells is no different than wild type (Additional file 1). Additionally the rate of clearing of bacteria from a suspension culture is also no different between the 3 strains (data not shown). This indicates that the difference in plaque size is not likely due to differences in the rates of phagocytosis of bacteria. 

**Table 1:** Comparison of plaque size on rich broth and minimal media plates

**Strain**	**Plaque area (mm**^**2**^**) on minimal medium plates**	**Plaque area (mm**^**2**^**) on rich broth plates**
WT	1.41 +/−0. 17	0.94+/−0.08
KO	0.28 +/− 0.04*	0.020+/−0.002*
OE	2.33 +/− 0.28*	0.018+/−0.002*

Cells were plated for 4 days on LP (minimal media plates) with *E.coli* B/r and for 11 days on 1/2xHL5 plates on *E.coli* B/r (rich broth plates). The areas of between 30 to 70 plaques per strain were determined on a minimum of 5 different plates per strain. Experiments were repeated at least 3 times with similar results on different days. Absolute plaque size differs with different batches of plates but the relative plaque size differences between strains are highly reproducible. Errors are standard error of the mean. * indicates difference is significant from Wt (p value < 0.001). There was no significant difference in plaque size between KO and OE (p value = 0.26) on rich broth plates but significant differences between KO and OE on minimal media plates (p value < 0.001).

**Figure 1:**
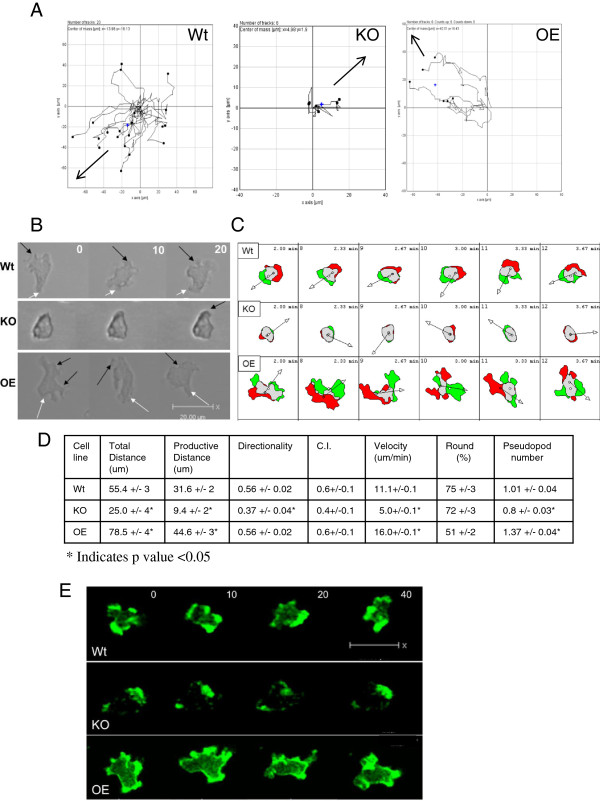
***ampA *null cells are defective migrating on top of agar; overexpressing cells move very rapidly. A**) Chemotaxis plots of individual cells in a representative field. Arrows indicate the location of the folic acid source. Note scale difference on the plot of *ampA* knockout. **B**) Morphology of cells migrating on top of agar. Black arrows indicate pseudopods; white arrows, uropods. Time is in seconds. Scale bars are 20 um. **C**) Difference plots created by overlaying the cell outline in frame 1 with the cell outline in frame 2. Green represents areas of protrusion and red represents areas of retractions. **D**) Quantification of cell migration. Data are the averages +/− the standard error of the mean. Differences were checked using a 2 tailed paired Student’s t-test with p < 0.05 indicating a significant difference (*). Data are compilations of 40–100 cells tracked in 6 different experiments. C.I. Chemotactic Index (see Methods). The distance migrated (total) is in 5 minutes. Productive distance is the Euclidian distance from the start of imaging to the final time point. Directionality is the ratio of productive to total distance and is a measure of how progressive the movement is. **E**) Differences in actin organization in *ampA* mutant cells migrating to folic acid on top of agar. Live cells carry a plasmid containing the ABD120-GFP fusion protein (a marker for F-actin). Wt and AmpA overexpressing cells contain the blasticidin resistant version of the plasmid and *ampA* null cells carry the G418 version of the plasmid. The direction of migration is toward the top of the image. Scale bar is 20 um.Time is in seconds.

In order to determine if the difference in plaque size might reflect a difference in cell motility, the migration of single cells toward folic acid was monitored. Growing cells were placed on a thin layer of agar and folic acid was spotted a millimeter away. The migration of cells to the folic acid spot was imaged by time lapse video microscopy [26]. The *ampA* null cells were largely unable to migrate toward folic acid (Figure 1A and D). They mostly remained stationary, occasionally reaching out a pseudopod, but almost never doing much more than rolling back and forth in the same spot (see movies in Additional files 2 (Wt) and 3 (KO)). This caused a decrease in velocity and distance moved when compared to wild type (Figure 1A and D). The few knockout cells that are actually able to migrate do sense the chemoattractant and migrate towards it, albeit much more slowly than wild type (Figure 1A). The low chemotactic index (a measure of how directly a cell migrates to the chemoattractant defined in methods) for the *ampA* null cells reflects the failure of most of the cells to migrate rather than a loss of directionality when they do migrate (Figure 1D). AmpA overexpressing cells move at a much increased velocity and cover much more distance than wild type (Figure 1A and D). Several cells were measured that moved at speeds up to 30 um per minute but the average velocity was 16 um/min as compared to 11 um/min for the wild type (Figure 1A and D & Movie in Additional file 4). Both Wt and AmpA overexpressing cells showed a similar ability to migrate directionally towards the folic acid as indicated by the chemotactic index. 

In comparing morphologies, wild type cells have a true pseudopod and uropod (Figure 1B). Knock out cells produce fewer pseudopods (Figure 1B). The pseudopods that the cells do extend are more rounded and not at all elongated as in wild type. In contrast, the overexpressing cells form multiple pseudopods, statistically more than wild type, and the cells are also much more elongated, as can be seen in the roundness value (Figure 1D). Roundness is the ratio between the width and the length of the cells. Perfectly round cells have a roundness value of 1 or 100%. Figure 1C illustrates the differences in protrusion and retraction of pseudopods. The protrusions are illustrated in green and retractions in red. Knock out cells form very small protrusions and retractions which use very little of their cell mass. In contrast, overexpressing cells use most of their cell mass when forming protrusions (Figure 1C).

### AmpA regulates the level of actin polymerization in growing cells

**Figure 2:**
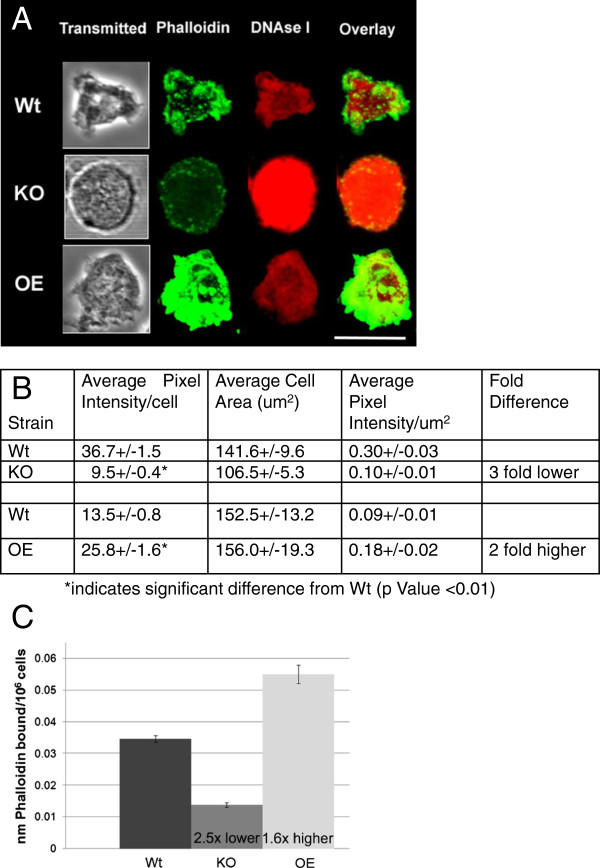
**AmpA influences the level of polymerized F actin. A**) Wild type, *amp*A knockout (KO) and overexpresser (OE) cells were stained with Alexa 488-phalloidin to detect F-actin (green) and TRITC labeled DNAse I to detect G-actin (red). Imaging conditions were optimized for levels of phalloidin staining (see Methods). The fields of cells from which the representative individual cells were excised are in Additional file 5. The images are 3D reconstructions from a confocal z series. Scale bar is 20 um. The fluorescent F-actin images were all equally enhanced to more clearly see the F-actin in the *ampA* knockout. **B**) Fields of Wt, KO and OE cells stained with Alexa 488 phalloidin shown in Additional file 5 were quantified (see Methods). The average pixel intensity per cell, cell surface area and pixel intensity/um^2^ are shown. Significance was determined by a two-tailed paired Student’s t-test with a p value < 0.05 indicated as significantly different from Wild type. 15 to 20 cells per strain were quantified per experiment. The staining experiment was repeated 6 different times with cells grown on different days. **C**) The amount of Alexa 488-phalloidin bound to cells was determined (see Methods). Results are averages of 3 different experiments with data points in triplicate. Error bars are standard error of the mean.

**Figure 3:**
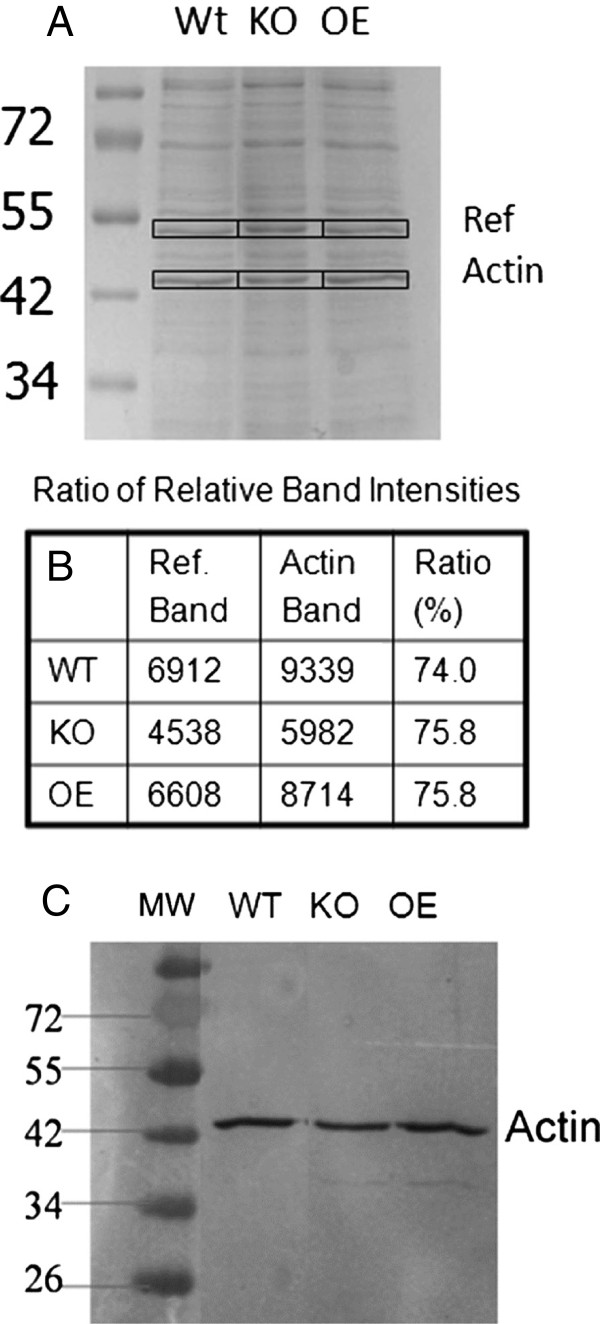
**AmpA does not change the amount of total actin protein in the cells. A**) Polyacrylamide gels were loaded with total protein from 1 x 10^5^ cells per lane and stained with Coomassie. **B**) The amount of protein loaded was determined to give a linear range signal when scanned on a densitometer. To control for protein loading differences the ratio of intensity of the F-actin band to a reference band was determined. **C**) Western blot of total protein using an anti-actin antibody. 1 x 10^5^ cells were loaded per lane. In control experiments this amount of protein gave antibody staining for actin which was linearly dependent on the amount of protein loaded.

The differences in pseudopod protrusion suggested the possibility that actin polymerization could be influenced by AmpA. In order to analyze the actin cytoskeleton, growing cells were stained with fluorescently labeled phalloidin and DNAse I to detect polymerized F-actin and unpolymerized globular actin (G-actin) respectively (Figure 2A, zoomed images of representative individual cells and Additional file 5A and 5B for whole fields of cells). *AmpA* null cells clearly contain significantly less polymerized F-actin than do wild type cells. By contrast, the overexpressing cells polymerize more actin than do wild type. Quantification of the images indicates that *ampA* null cells have about 3x less F-actin than wild type while AmpA overexpressers have about 2x more F-actin than wild type (Figure 2B). In order to confirm that there was a difference in F-actin levels as a function of *ampA* expression, the amount of phalloidin binding to cell extracts was also measured (Figure 2C). This more accurate method indicates that *ampA* null cells have 2.5x less F-actin than wild type while AmpA overexpressers have 1.6x more F-actin. In order to determine if the difference in F-actin level is due to actin polymerization rather than actin protein synthesis, the total amount of actin protein in the 3 cell lines was compared in two different ways (Figure 3A, B, and C). First, equal numbers of cells were harvested and subjected to polyacrylamide gel electrophoresis and stained with Coomassie blue. The amount of protein loaded on the gels was determined to be in a linear range for the actin protein band. The relative amount of actin protein was quantified. In order to control for differences in loading, a ratio of actin protein to a reference band was determined. These ratios were identical for all three cell lines indicating that the same amount of total actin protein was present (Figure 3A and B). This result was confirmed by western blots which also showed no significant difference in the amount of total actin protein in the 3 cell lines, indicating that AmpA controls the levels of actin polymerization in growing cells, not the amount of actin protein (Figure 3C). 

In order to observe the effects of the *ampA* mutations on the actin cytoskeleton in live cells, Wt, *ampA* null and AmpA overexpressing cells were transfected with a plasmid, GFP-filABD, which contains the actin binding domain of the ABD120 protein fused to GFP [27,28]. While migrating *ampA* null cells are more rounded, they clearly show actin polymerizing directionally in polarized, pseudopod and uropod like structures although they are not as large and extended as seen with Wt or AmpA overexpressing cells (Figure 1E). They do not show the pseudopod splitting that seems more prevalent in growing cells as they migrate to folic acid. By contrast, the AmpA overexpressing cells not only show more pseudopod splitting but they also show actin polymerized strongly around most of the entire cell cortex. The Wt and AmpA overexpressing cells carry a GFP-filABD plasmid that contains a blastocidin resistance cassette. The *ampA* null cells had to be transformed with a GFP-filABD plasmid that carried a G418 resistance marker that is usually present at much higher copy numbers. The F-actin level in the knockout cells was too low for detection when the blasocidin cassette was used as a selectable marker. Thus it is not possible to compare differences in actin levels in this image, only actin distribution and dynamics.

### AmpA influences cell migration in an environment dependent manner

Loss of AmpA clearly reduces actin polymerization and cell migration, while overexpressing AmpA results in rapid migration and excessive actin polymerization. The differences in cell migration are clearly consistent with the differences observed in plaque size that we had reported [25 and summarized in Table 1. When analyzing plaque morphology one normally uses a low nutrient agar (minimal media plates) that reduces the density of the bacterial lawn and allows plaques to spread. It is under these conditions that AmpA overexpressing strains make much larger than normal plaques. However if one wants to screen large numbers of *Dictyostelium* plaques on bacterial plates, a higher nutrient agar is used and the bacterial lawn that forms is denser and the plaques are much smaller [29]. Surprisingly under these conditions AmpA overexpressing cells form plaques that are much smaller than wild type and are about the same size as those formed by the *ampA* null cells (Table 1 and Additional file 6B insets). In order to better understand the role of AmpA in influencing plaque size in high density lawns, cells at the plaque periphery were imaged at high magnification from underneath the agar plates and the migrations of individual cells that could be distinguished at the plaque periphery were tracked (Additional file 6A and B). Increasing the density of the lawn of bacteria appears to inhibit the ability of AmpA overexpressing cells to penetrate the bacterial lawn. Analysis of the centroid tracks of individual cells at the plaque periphery indicates that both wild type and *ampA* null cells move directly perpendicular to the edge of the plaque, but, surprisingly, the overexpressing cells migrate circumferentially around the plaque (Additional file 6B). The yellow lines mark the tracks of individual cells over 30 minutes. The overexpressing cells travel nearly twice as fast as the wild type cells and cover more distance even though it is in a circumferential direction (Additional file 6A and B). By traveling circumferentially around the plaque the overexpressing cells avoid the problem of penetrating into the dense lawn of bacteria. The *ampA* null cells, by contrast, cover about the same total distance as the wild type cells but they cover significantly less productive distance (Additional file 6A). The productive distance moved by the *ampA* null cells is only about 40% of the total distance migrated, indicating that most of the movement of the null cells is rolling back and forth rather than progressively moving out into the bacterial lawn (Additional file 6A). Regardless of the density of the bacterial lawn, both wild type and overexpressing cells still migrate efficiently; what differs is their direction of travel. The smaller plaques of the overexpressing strains under rich broth conditions are clearly a result of the failure of the cells to enter the bacterial lawn while the small plaque size of the *ampA* null cells is the result of multiple reversals in direction so that the cells cover less productive distance. This raises the question of whether over expression of AmpA results in a reduction of substrate adhesion. Such a defect might permit faster migration but prevent the cells from being able to adhere strongly enough to the substrate to be able to exert the force to migrate through the dense lawn of bacteria.

### AmpA influences cell-substrate adhesion

**Figure 4:**
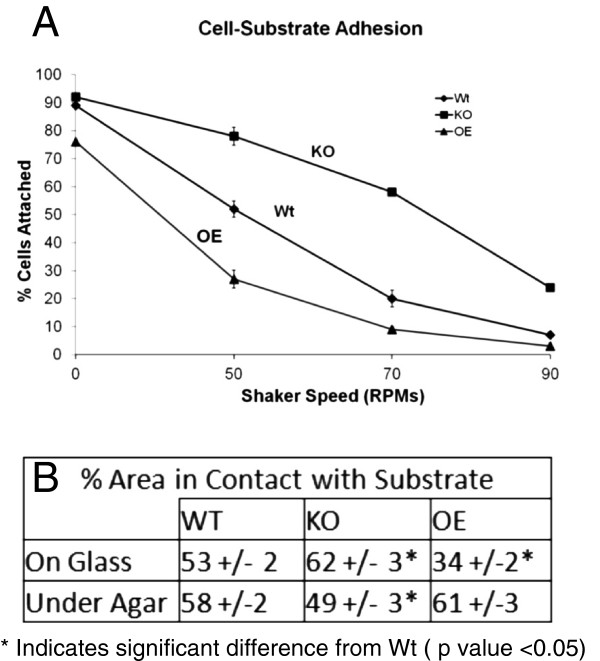
**AmpA influences cell substrate adhesion and substrate contact area. A**) AmpA influences cell substrate adhesion. Cells grown on petri dishes were shaken at increasing shaker speeds, RPM (rotations per minute) for 45 minutes and the percent of cells remaining on the dish was determined. **B**) AmpA influences the % of the cell surface in contact with the substrate. To determine the percentage of the cell in contact with the substrate cells were imaged using transmitted light and in reflection mode to identify the area of the cell in contact with the substrate (Additional file 7 for raw data). The area of the cell and the area of the contact sites were determined and the contact sites were expressed as a % of the total area of the cell. The number of cells imaged was 65 to 125 for cells sitting on glass and 50 to 95 for cells migrating under agar. Results are averages from two different experiments preformed on different days. Error bars are the standard error of the mean. * indicates a significant difference with a P value < 0.05 using a two tailed paired Student’s t-test.

In order to address the question of whether the differences in migration detailed above were due to an effect of AmpA on cell substrate adhesion, the relative ability of *ampA* null and AmpA overexpressing cells to adhere to a substrate was determined. The cells were allowed to adhere to culture dishes overnight. They were then shaken at increasing shaker speeds and the percentage of cells that remain attached after 45 minutes at each speed was determined (Figure 4A). At 50 rotations per min (rpms) 80% of the *ampA* null cells remain attached to the substrate, while only 50% of Wt cells remain attached. By contrast, less than 30% of the AmpA overexpressing cells are still adhering to the substrate. Thus, *ampA* knockout cells are clearly more adherent than wild type cells, while the AmpA overexpressing cells have significantly decreased adhesion. Reflection imaging was used to determine the percent of the cell area that was in contact with the substrate. For wildtype cells growing on glass cover slips about 50% of the cell surface area was in contact with the substrate but for AmpA overexpressing cells far less, 34%, was in contact with the substrate. For *ampA* null cells, more of the cell surface area, 62%, was in contact with the substrate than for wild type cells (Figure 4B top row of table (on glass); transmission and reflection images in Additional file 7A). 

### Migration under agar rescues the motility defect of *ampA* null cells and reduces the rapid migration of AmpA overexpressing cells

**Figure 5:**
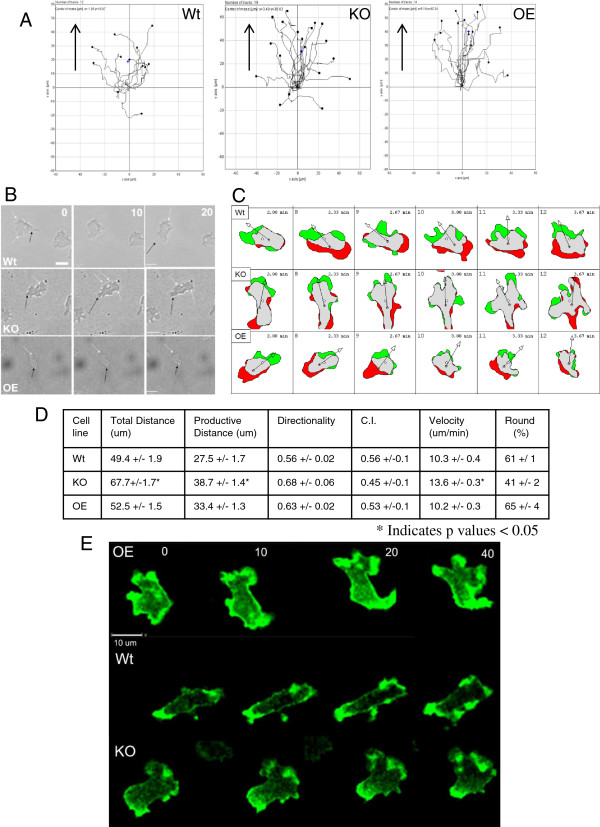
***AmpA *null cells migrate better than wild type under agar; overexpressing cells show reduced migration.** Cells were allowed to migrate to folic acid under agar as described in methods. Images were acquired every 10 seconds for 5 min. **A**) Chemotactic plots are a representative field. Arrows indicate the location of the folic acid source. **B**) Morphological differences between the cell types under agar. Black arrows represent pseudopods; white arrows indicate uropods. Time is in seconds. Scale bar is 20 um. **C**) Difference plots were created by overlaying the outline of a cell in frame 1 with its outline in frame 2. Green represents areas of protrusion and red represents areas of retractions. **D**) Quantification of migration under agar on glass. The data are the averages +/− the standard error of the mean. Differences were checked for significance (*) using a 2 tailed paired Student’s t-test, p < 0.05 was deemed significant. Data are the compilations of 90–120 cells tracked in 6 experiments. The distance migrated is in 5 minutes. **E**) Migrating under agar on glass *ampA* knockout cells form true pseudopods and uropods. Live cells carrying the ABD-GFP plasmid were placed in a well cut in 0.8% agar opposite a well containing 0.5 mM folic acid. Cells were allowed to sense the gradient for 4–5 hours before imaging on a Leica SP5 confocal microscope. Time is in seconds. Scale bar is 10 um. The direction of migration is to the top of the image. Wt and overexpressing cells contain the blasticidin resistant version of the plasmid and *ampA* null cells carry the G418 version of the plasmid.

Since AmpA overexpressing cells appear to have trouble penetrating a lawn of dense bacteria and show less adhesion to the substrate, we tested the ability of AmpA mutant cells to migrate in an environment where they have to migrate under agar, which requires more force [30]. Cells were placed in a well in a thin layer of agar on a glass cover slip about 1mm from a well containing folic acid. Over time the cells slip under the agar and migrate on the glass cover slip towards the folic acid. In this environment cells have a layer of agar on top of them to which they can adhere and form contacts and they migrate on a less deformable and less adhesive glass cover slip. Under these conditions the *ampA* knockout cells no longer have any migration defect (Figure 5A and D and movies in Additional files 8 (Wt) and 9 (KO). *AmpA* null cells actually move significantly better than wild type cells and even better than overexpressing cells under these conditions. Their velocity increased significantly over wild type reaching an average of 13 um/min (Compare Figure 1D with 5D). The distance traveled also increased significantly compared with wild type. In contrast, the AmpA overexpressing cells appear to revert to the wild type phenotype (Movie in Additional file 10). There is no significant difference between wild type and overexpressing cells in any of the migration parameters measured (Figure 5A and D). 

Migration under agar on glass also produces significant changes in morphology and substrate contact. Knockout cells under agar on glass now have true pseudopods and uropods (Figure 5B and C) and are more elongated (Figure 5D). This is particularly apparent when one compares the roundness value of 72% for the *ampA* null cells migrating on top of agar with the roundness value 41% when migrating on glass under agar (Figure 1D vs 5D). Additionally, the number of pseudopods that knockout cells extend is significantly greater than wild type. These differences in the morphology of the *ampA* null cells migrating on glass under agar are also apparent when one observes the actin cytoskeleton in live migrating cells under agar (Figure 5E). The *ampA* null cells migrating under agar show clear dynamic pseudopods that appear to split frequently. For the AmpA overexpressing cells, actin remains polymerized around the entire cell cortex (Figure 5E) although difference plots show less pseudopod extension and retraction than was seen when they migrated on top of agar (Figure 5C).

The difference in migration behavior of *ampA* mutant cells under these different environmental conditions raises the question of whether either the difference in the substrate or the presence of agar overlaying the cells alters the contact of the cells with the substrate. Reflection imaging was used to compare the percentage of the ventral cell surface in contact with the cover slip in the cells migrating under agar (Figure 4B bottom row of Table (under agar); transmission and reflection images in Additional file 7B). The wild type cells show relatively the same percentage of the cell area in contact with the substrate in both conditions (53% sitting on glass vs 58% migrating under agar). By contrast the *ampA* mutant strains both show significant changes in substrate contact area under the two different environmental conditions. The AmpA overexpressers show a significant increase in % of surface area in contact with the substrate when migrating under agar on glass than when sitting on top of glass (34% sitting on glass and 61% migrating under agar). The *ampA* knockouts show significantly less of their surface in contact with the substrate when migrating under agar than when sitting on glass (49% in contact when migrating under agar vs 62% sitting on glass in media). Migration at an air-water interface on top of agar represents a very different environment than migrating under agar on a glass cover slip. In order to test whether the substrate influences the rate of migration of the cells, we compared two more similar conditions; migration under agar on a glass cover slip with migration under agar on a plastic Petri dish substrate.

### AmpA overexpressing cells migrate more rapidly under agar on plastic than they do on glass and *ampA* null cells migrate more poorly

Cells were induced to migrate under agar as before but on plastic cell culture dishes rather than glass cover slips. Under these conditions *ampA* null cells now migrate identically to the wild type, moving with speeds and distances that are the same as wild type rather than faster as they did on glass under agar (Table 2). Wild type cells slowed a little bit from 10.3 um/min to 8.3 um/min but knockout cells slowed much more; from 13.6 um/min on glass under agar to 8.1 um/min on plastic. This result is statistically significant with a p value of < 0.005. Knockout cells migrate quite well, however, so the phenotype seen when migrating on top of agar is still rescued when migrating under agar on plastic but they do not migrate faster like they do when the substrate is glass. The biggest change is seen with the AmpA overexpresser. While knockout and wild type cells slowed down relative to their rates on glass, overexpressing cells actually increased their velocity significantly on plastic by about 20% from migration over glass. It is also interesting to note that while overexpressing cells moved faster on plastic then glass, these rates are still much slower, by about 40%, than their rates on top of agar. Thus the amount of AmpA clearly influences cell migration in a substrate and environment dependent manner. Excess AmpA clearly provides an advantage on soft substrates like agar enabling cells to migrate more rapidly, while loss of AmpA favors cells migrating on hard surfaces like glass. 

**Table 2:** The substratum plays a role in the effect of AmpA on cell migration

**Cell line**	**Total distance (um)**	**Productive distance (um)**	**Directionality**	**C.I.**	**Velocity (um/min)**	**Roundness (%)**	**Pseudopod number**
Wt	41.2 +/− 1.6	28.2 +/− 1.2	0.7 +/− 0.02	0.5 +/−0.1	8.3 +/− 0.5	47 +/−3	1.44 +/− 0.03
KO	39 +/− 2	24.5 +/− 1.3	0.7 +/−0.02	0.5 +/−0.1	8.1 +/− 0.5	57+/−2*	1.72 +/− 0.03*
OE	61 +/− 3*	39.2 +/− 1.9*	0.7 +/− 0.03	0.5 +/−0.1	12.2 +/− 0.6*	64+/−2*	1.45 +/− 0.03

Cells were placed in wells cut in a thin layer of 0.8% agar in a plastic dish. Folic acid was placed in another well. The cells were allowed to sense the gradient for 4–5 hours before imaging every 10 seconds for 5 min. Data are the averages +/− the standard error of the mean. * indicates significant difference from wild type. Differences were checked for significance using a 2 tailed Student’s t-test p<0.05. Data are the compilations of 40–50 cells tracked over plastic under agar. The distance migrated is in 5 minutes. Terms are defined in the legend to Figure 1 and in methods. AmpA overexpressing cells move significantly farther and faster than wild type, but their directionality and chemotactic index are unchanged. *AmpA* knockout cells move in a statistically similar manner to wild type and not faster as they did on the glass substrate.

### AmpA protein is localized in punctuate membrane vesicles and in a perinuclear compartment

In order to better understand the mechanism by which AmpA influences actin polymerization, substrate adhesion, and cell migration, AmpA fusion protein constructs were generated in order to use immunofluorescence microscopy to determine the location of AmpA in the cell. Strains were made that contained AmpA with a TAP Tag fused to its C-terminus (Additional file 11A) [31]. Two strains containing the AmpA-TAP fusion protein were created. The first AmpA-TAP tag strain was created by electroporating the entire circularized plasmid into wild type cells. This led to the AmpA-TAP tag fusion protein being expressed on an extrachromosomal plasmid and resulted in a strain that had an AmpA overexpresser phenotype. It made large plaques on bacterial lawns (Additional file 11C and D). Like the AmpA overexpresser strains previously characterized it arrested development at mound stage (Additional file 11E, compare to AmpA overexpresser strains in [19] Figure 10F and [21] Figure 3A). We call this strain AmpA-Tap tag-OE, for overexpresser phenotype. The second strain was created by introducing a linearized KpnI-NotI DNA fragment of the AmpA-Tap tag vector into wild type cells. This fragment contained only the *ampA* gene fused to the Tap tag plus the blastocidin resistance cassette. While this fragment did not integrate into the *ampA* gene and create a gene replacement, it did result in a cell line that behaved as a wild type cell line, contained about 3x less AmpA-Tap tag protein that the stain with the overexpressing phenotype (Additional file 11B) and formed plaques on bacterial lawns that were not significantly different from the size of Wt plaques (Additional file 11C and D). Like Wt, this strain progressed normally through development (Additional file 11E). We refer to it as AmpA-Tap tag-Wt.

We also constructed a second vector containing an AmpA fusion protein. This one had the mRFP protein fused to the N terminus of the AmpA protein immediately after the hydrophobic leader sequence (Additional file 12A) [32]. When this plasmid was introduced into wild type cells as a circular plasmid, the cells also displayed an AmpA overexpresser phenotype, making large plaques on bacterial lawns and arresting development at mound stage (Additional file 12B, C and D). The fact that both overexpressing fusion protein vectors showed the typical AmpA overexpression phenotype indicates that both the AmpA-tap tag and the mRFP-AmpA fusion proteins are active and functional and can thus be used as probes to localize AmpA. Initial localization experiments were done with the AmpA-Tap tag constructs but later batches of anti-tap antibody were not suitable for immunofluorescence studies so the mRFP-AmpA construct was used instead. Where possible, results are shown with both constructs.

**Figure 6:**
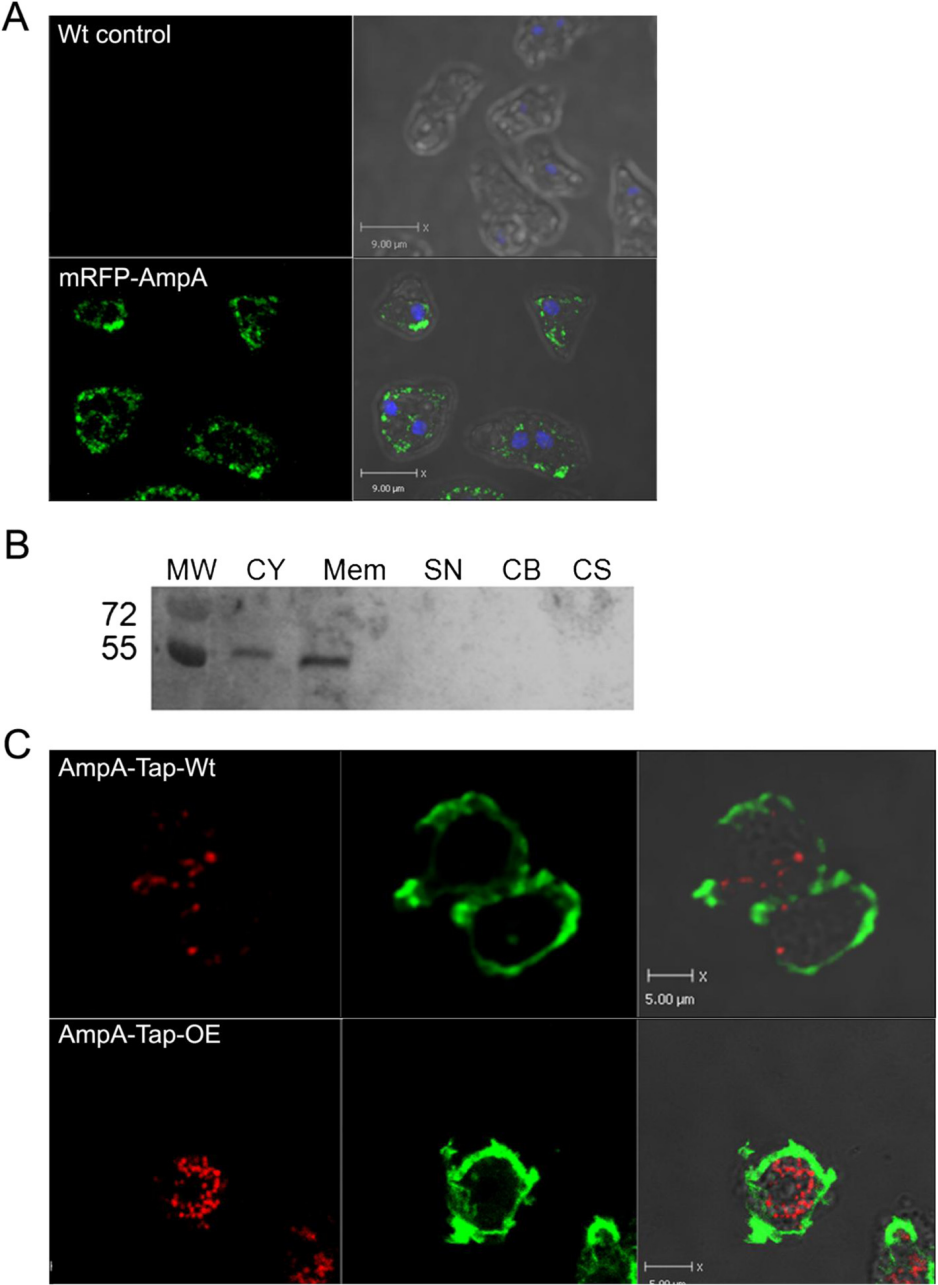
**AmpA is found in vesicles and in the perinuclear region but not with F-actin. A**) Wt control cells (top row) and cells carrying the mRFP-AmpA fusion protein construct (bottom row) were immunostained with rat anti-RFP antibody and goat anti-rat antibody conjugated to Alexa Fluor 488 (Invitrogen). The left panel is anti-RFP antibody staining (green), and the right panel shows overlays of the immunostaining on DAPI stained images (blue) to mark the nucleus and transmitted images. The scale bar is 9 um. **B**) Western blots of cell fractionations of mRFP-AmpA expressing cells. MW is molecular weight standard. CY-Cytoplasm, Mem-Membrane, SN-Soluble Nuclear, CB-Chromatin Bound, CS-Cytoskeletal. Controls for this fractionation are in [25]. **C**) AmpA does not colocalize with F-actin. Cells were fixed and stained for AmpA Tap tag and F-actin. Green: Alexa fluor 488 phalloidin stain for F-actin; Red: AmpA Tap tag antibody. Representative images are single optical sections from a Z series. Scale bar is 5 um. An AmpA-Tap-Wt (top) and an AmpA-Tap-overexpressing strain (bottom) are shown.

Both AmpA-Tap tag-OE and AmpA-Tap tag Wt cells and cells containing the mRFP-AmpA fusion protein construct were imaged using fluorescently labeled anti-tap tag or anti-RFP antibodies (Figure 6A showing the mRFP-AmpA construct and Additional file 13A showing AmpA-Tap tag Wt and OE). AmpA protein was localized to a series of punctuate spots present throughout the cell and also in a perinuclear location which is evident in Figure 6A where the nuclei are stained with DAPI. All three strains showed the same pattern of AmpA location indicating that neither the source of the fusion protein or the overexpressing phenotype appeared to influence the location. The punctuate spots are likely membrane bound vesicles because cell fractionation studies show that AmpA is largely present in the membrane fraction (Figure 6B and Additional file 13B showing the AmpA-Tap tag construct). A small amount is found in the cytoplasm but since AmpA has a hydrophobic leader sequence characteristic of secreted proteins it is likely that this is due to rupture of some of the vesicles during fractionation. 

### AmpA does not colocalize with sites of actin polymerization but a portion of the AmpA protein is located in the Golgi and an ER derived compartment that is perinuclear

Because of the strong affect AmpA has on actin polymerization, it is possible that vesicles containing AmpA localize to sites of actin polymerization or strong actin staining. AmpA-TAP-tag labeled cell lines with both wild type and overexpresser phenotypes were stained with phalloidin and the anti-tap tag antibody. Figure 6C shows representative optical sections from both of the AmpA TAP tag fusion protein containing cell lines. The optical sections show that actin is present at the edges of the cell and that AmpA is localized in punctate spots or vesicles throughout the cell, but there is no true overlap between the two, other than by chance.

**Figure 7:**
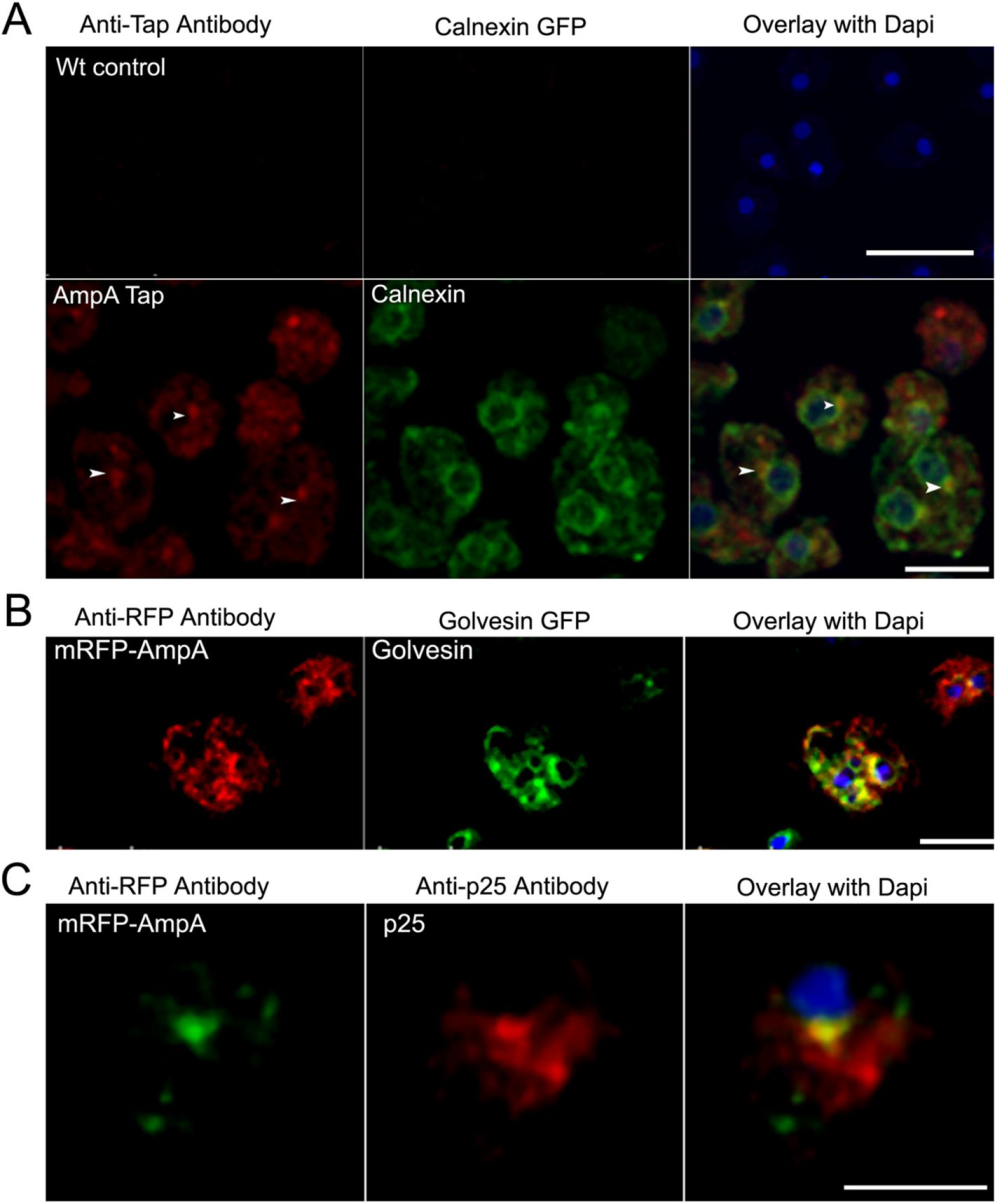
**Colocalization of AmpA with calnexin, golvesin and p25. A**) Some AmpA is found in a perinuclear compartment and colocalizes there with the ER marker calnexin. Top panels are Wt control cells that do not contain the calnexin GFP or the AmpA tap tag fusion protein. The cells were fixed, permeabilized and stained with rabbit anti-TAP antibody and goat anti-rabbit antibody conjugated to FITC (red). The second row panels show AmpA tap tag cells containing calnexin GFP (green) fixed, permeabilized and stained with rabbit anti-TAP antibody and goat anti-rabbit antibody conjugated to FITC (red). Cells were incubated with DAPI to stain the nuclei (Blue). Arrows indicate areas of calnexin and AmpA tap tag colocalization in a perinuclear compartment. Scale bars are 18 um for the field of cells in top row and 9 um for the zoomed images in the second row. Images are single optical sections from a confocal z-series. **B**) AmpA is found in the Golgi colocalizing with N-golvesin. mRFP-AmpA cells were transformed with a plasmid carrying N-Golvesin-GFP (Golgi marker-green) incubated, fixed and stained with rat anti-RFP antibody followed by Alexa goat anti-rat antibody (red). Arrow indicates the area of colocalization in the Golgi. DAPI (blue) stains the nucleus. Images are single optical sections from a z-series. Scale bar is 11um. **C**) AmpA colocalizes with p25, a recycling endosome marker in a perinuclear compartment. mRFP-AmpA cells were stained with rat anti-RFP (green) and mouse anti-p25 (red). DAPI (blue) stains the nucleus. Arrow indicates area of p25 and AmpA colocalization. Images are single optical sections from a z-series. Scale bar is 11 um.

Since AmpA has a hydrophobic leader and must travel through the endoplasmic reticulum (ER), the hypothesis was that these punctuate spots were membranes from the ER or Golgi. To test this theory, AmpA-TAP tag cells were transformed with a plasmid containing Calnexin-GFP. Calnexin is a Ca^++^ binding protein found in the ER [33]. AmpA-TAP does colocalize with calnexin but only in a perinuclear compartment and not throughout the rest of the ER (Figure 7A). The average Pearson coefficient for localization throughout the whole cell is 0.271 (out of a possible 1.0 for complete colocalization) indicating the partial nature of this colocalization. 

N-golvesin GFP is a fusion protein that is found distributed in the membranes of the Golgi and Golgi derived vesicles including endosomes and contractile vacuoles [34]. mRFP-AmpA cells were transformed with the N-golvesin-GFP containing plasmid. AmpA was seen to colocalize with N-golvesin predominantly in the Golgi adjacent to the nucleus (Figure 7B). It also showed some colocalization with AmpA in vesicles near the perimeter of the cell but there were other golvesin labeled vesicles that did not contain AmpA (Figure 7B). The Pearson coefficient for this localization throughout the whole cell is 0.23 indicating the partial nature of the colocalization sites. Thus AmpA is in the Golgi and in some Golgi derived vesicles near the cell periphery.

### AmpA colocalizes with the endosomal recycling marker p25 in a perinuclear compartment

Since AmpA is found in a perinuclear compartment and in vesicles, we looked to see if it was associated with other endosomal markers. The protein p25 has been used to identify a perinuclear slow endosomal recycling compartment [35]. AmpA-mRFP colocalizes with p25 in the perinuclear region (Figure 7C). The average Pearson coefficient is 0.216 indicative of the colocalization of these proteins in some compartments but not all compartments. This raises the question of whether AmpA colocalizes with p25 because it plays a role in membrane recycling or because AmpA itself is recycled from the membrane.

### AmpA is localized at low levels on the cell periphery and recycled via endocytosis

**Figure 8:**
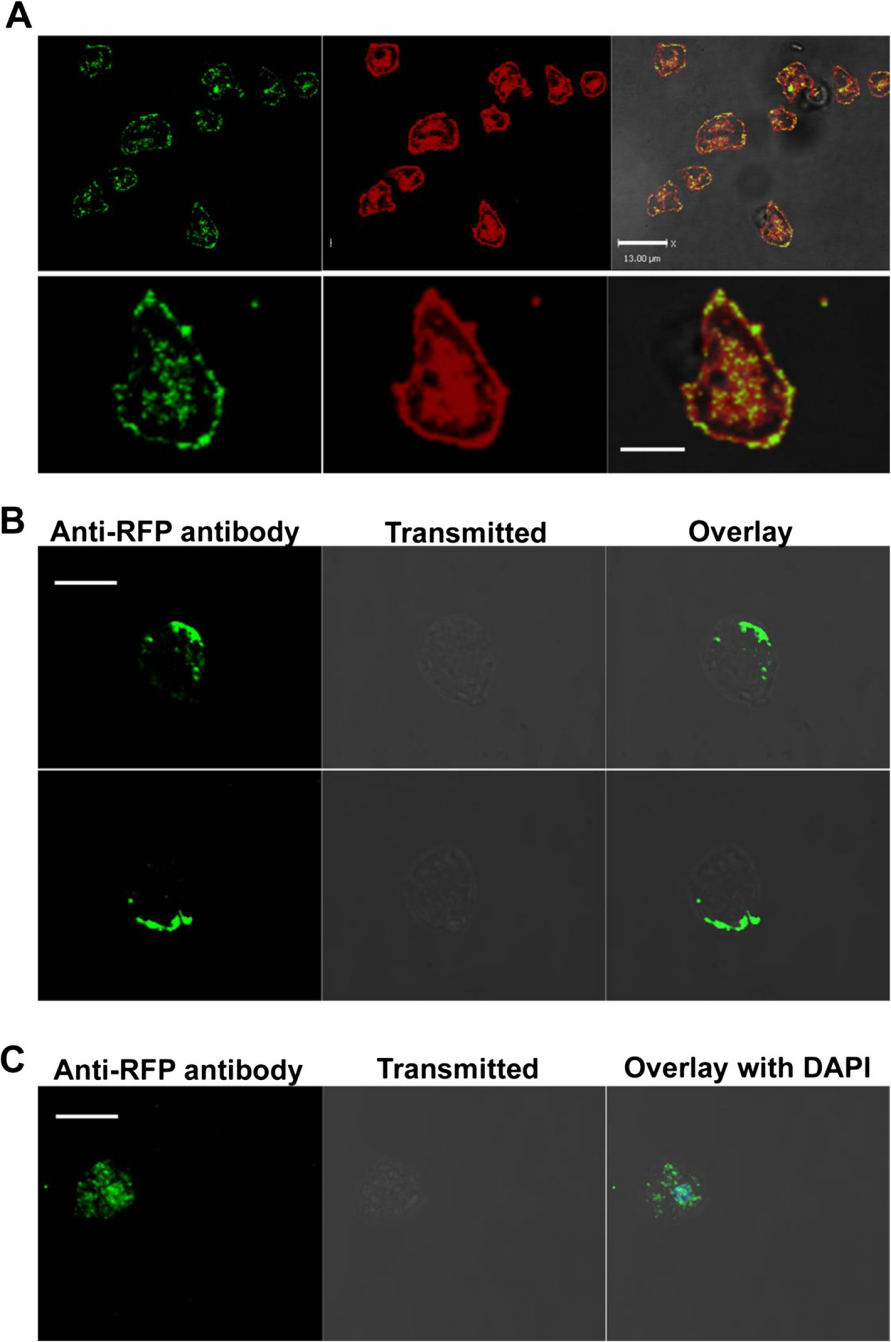
**Some AmpA protein is found extracellularly on the cell surface. A**) Live cells were incubated with the membrane stain DiI and with rat anti-RFP antibody in the dark at room temperature. Cells were washed and then incubated for 1 hour with goat anti-rat antibody conjugated to Alexa-Fluor 488, then washed and fixed but never permeabilized. Images represent single optical sections of a z-series. Arrows indicate colocalization of mRFP-AmpA with DiI at the cell periphery. Scale bar is 13 um in the field images (top row) and 5 um in the zoom of a single cell (bottom row). **B** and **C**) AmpA on the cell surface is endocytosed and traffics to a perinuclear endosomal recycling compartment. Live cells were incubated 4°C with rat anti-RFP antibody. **B**) Some of the coverslips were maintained at 4°C to prevent endocytosis, washed to remove excess anti-mRFP antibody, fixed and then permeabilized and labeled with goat anti-rabbit second antibody. Slices of cells from a Z series to show that the labeling is largely on the outside of the cell and not in the interior. Scale bar is 10 um **C**) Cells on the other coverslips (panels labeled 22 degrees) were washed to remove excess anti-mRFP antibody, then incubated at room temperature for 15 min to allow endocytosis of the antibody bound mRFP-AmpA and then fixed and permeabilized to allow labeling with second antibody to detect anti-RFP antibody that had been internalized with the AmpA. The image is a slice from a Z series. The scale bar is 10 um.

Since AmpA is found in a cell compartment consistent with a role in membrane protein recycling, it is possible that AmpA would be found on the plasma membrane. In standard fixation procedures we have not clearly seen AmpA on the membrane but the association could be lost due to the fixation or permeabilization. To determine if AmpA is on the membrane, live AmpA mRFP cells were incubated with DiI membrane stain and then with anti mRFP primary antibody either at room temperature (Figure 8A) or at 4°C (Figure 8B). The cells were then washed and incubated with goat anti rabbit secondary antibody conjugated to FITC. The figures represent optical sections from a z-series and indicate that under these conditions where live cells were used, AmpA colocalizes with DiI on the membrane. These results suggest that AmpA is on the plasma membrane but, when cells are permeabilized prior to staining, this fraction of AmpA is lost, perhaps indicating a weak interaction. Although these cells were never permeabilized, there is a fraction of labeled intra-cellular AmpA which is likely the result of endocytosis of the extracellular cellular AmpA plus bound antibodies (Figure 8A). The internalized AmpA is in the same locations as the DiI which enters the live cells by endocytosis (Figure 8A). The internal fraction of AmpA staining is largely missing in Figure 8B where the live cells were maintained at 4°C to prevent endocytosis. 

In order to determine if AmpA is being endocytosed, live AmpA-mRFP cells were incubated with primary anti mRFP antibody at 4^o^ for 10 minutes [35]. Some of the live cells were immediately incubated with secondary antibody and imaged (Figure 8B). These live cells showed mRFP-AmpA on the cell surface. A second set of the live cells were then incubated at room temperature for 15 minutes to allow time for the AmpA-mRFP plus antibody to be endocytosed. These cells were then washed and fixed with formaldehyde. The cells were then permeabilized to allow entry of the secondary antibody. This led to high intra-cellular staining of mRFP-AmpA, which could only happen if AmpA had been endocytosed by the live cells while bound to the primary antibody (Figure 8B and C). Some of this staining is located in the perinuclear area suggesting that endocytosed AmpA may travel to the perinuclear slow recycling complex where it was observed to colocalize with p25 (Figure 7C).

### AmpA overexpression increases endocytosis

**Figure 9:**
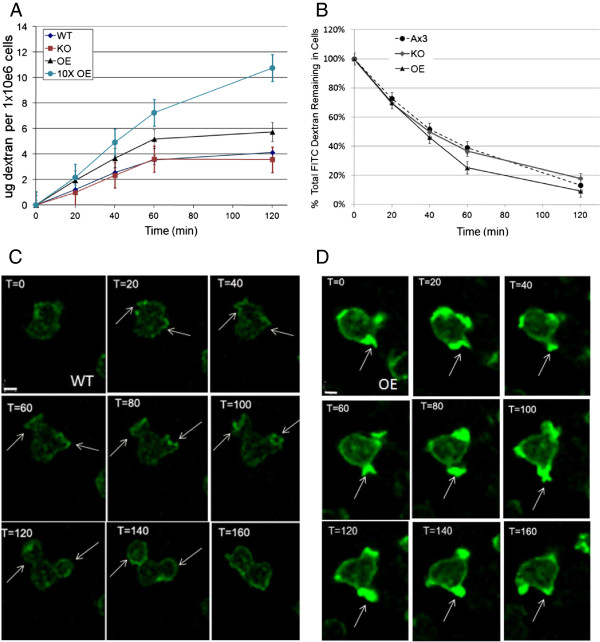
**AmpA overexpression increases the rate of endocytosis but not exocytosis.A**) FITC dextran was added to cells in shaking culture to a final concentration of 2 mg/ml. Aliquots were taken every twenty minutes and prepared as described in methods. Fluorescence was read on a Bio-Rad fluorimeter. **B**) Cells in shaking culture were incubated in FITC dextran as in **A**. They were then washed to remove extracellular FITC. Aliquots were taken every twenty minutes. The amount of FITC dextran present at Time = 0 was labeled as 100% for each cell type, and the FITC dextran remaining in the cell was taken as a percentage of the original amount**.** For **A** and **B**, error bars represent the standard error of the mean. The data represent the averages of a minimum of 5 separate determinations done on different days. **C and D**) Overexpression of *ampA* causes multiple endocytic cups to form repeatedly at the same site. **C**) Wt and **D**) AmpA overexpressing cells containing the blasticidin resistant ABD-GFP plasmid were placed in chambered cover slips overnight. Images were taken every 20 seconds for 5 minutes. These images are representative slices from the time courses. Calibration bar = 6 um. Images of additional AmpA overexpressing cells undergoing endocytosis are shown in Additional file 14.

Actin plays an important role in endocytosis and the fact that AmpA appears to cycle from the cell surface to interior vesicles and a perinuclear site raises the question of whether AmpA is passively endocytosed or whether it influences macropinocytosis. In order to determine if there was a change in levels of macropinoctyosis in AmpA mutants, the rate of FITC dextran uptake was measured. AmpA over expressing cells endocytose dextran at a more rapid rate than do the wild type cells (Figure 9A). The rate of endocytosis for the *ampA* knockout was not reproducibly different than wild type although in some experiments the rate does not plateau, in all other measures of endocytosis, such as time lapse videos or imaging of the amount of dextran in the cells (data not shown), the *ampA* null cells were similar to wild type. The AmpA overexpressing strain (OE1) makes about 3X the wild type level of AmpA protein [19]. A second AmpA overexpressing strain (OE2) that makes about 6X the wild type level of AmpA was created by selecting for an AmpA overexpresser that could grow in 10x the normal amount of G418 [19]. The rate of endocytosis by the OE2 AmpA overexpresser was even more rapid than that of the OE1strain. The OE1 strain endocytosed dextran at a rate of 5 ug dextran per 10^6^ cells per hour while the OE2 strain endocytosed the dextran at 7 ug per 10^6^ cells per hour compared to the wild type rate of 3.5 ug dextran per 10^6^ cells per hour. Thus, while AmpA is not essential for normal rates of endocytosis, overexpressing AmpA protein significantly increases the rate of endocytosis in a dose dependent manner. The rate of exocytosis, by contrast, was similar for all cell lines tested (Figure 9B). 

In order to understand the mechanism by which overexpressing AmpA protein increases the rate of endocytosis, live cells containing the ABD-GFP plasmid were imaged as they underwent endocytosis. The overexpressing cells showed a very unusual phenotype. They did not appear to make more endocytic cups but instead a number of the cells extended multiple endocytic cups from exactly the same point, one right after the other (Figure 9D versus wild type in Figure 9C and Additional file 14 for images of 2 additional AmpA overexpressing cells and the movies in Additional files 15 (Wt endocytosis) and 16 and 17 (AmpA overexpresser endocytosis)). In the wild type cells, the endocytic cup opens, engulfs the medium, and then retracts. At this point the polymerized actin at the site of cup formation is removed (Figure 9C and movie in Additional file 15). In a number of AmpA overexpressing cells, the endocytic cup opens, engulfs, then partially retracts, then opens and engulfs again appearing to use the same nucleus of polymerized actin to form the next endocytic cup (Figure 9D, Additional file 14 for 2 additional cells and the movies in Additional files 16 and 17). This repeated formation of endocytic cups at the same site is seen in 49% of the overexpressing cells (94 cells counted) but in only 8% of the wild type cells (98 cells counted). Other aspects of the endocytic process in the AmpA overexpressing cells seem entirely normal. The acidification of the endosomes occurs normally (Additional file 18), indicating that the actin surrounding the early endosome is properly depolymerized allowing the fusion of the early endosomes with the vesicle proton pumps.

When measuring endocytosis rates in the AmpA overexpressing cells, centrifugation of these cells prior to the assay led to a long delay before the cells were able to take up the dextran. For this reason we determined endocytosis rates by adding dextran to the media. The sensitivity of the AmpA overexpressing cells to either the cold or centrifugation itself led to the question of whether there was a contractile vacuole defect in the AmpA overexpressing cells but this does not appear to be the case. The contractile vacuole network in the Wt, *ampA* null and over expressing cells appeared to be identical (Additional file 19).

## Discussion

AmpA has effects on cell adhesion, cell migration, actin polymerization, and endocytosis. The question becomes how a protein not localized to sites of actin polymerization can play a role in these diverse cytoskeleton associated events. It is possible that AmpA acts as a signaling molecule that triggers these diverse events. Another possibility is that AmpA is involved in endocytosis and plays a role in membrane recycling. AmpA colocalizes with the p25 protein in the perinuclear region. Not much is currently known about the p25 protein other than that it is involved in the endosomal recycling pathway [35]. The identification of this protein was the first time recycling endosomes had been demonstrated in *Dictyostelium*.

Endosomal recycling has been extensively studied in mammalian cells. Some of the most well studied cases of recycling to the plasma membrane involve integrins. In order for migration to occur, integrins must be removed from the plasma membrane via endocytosis and then recycled back to form new adhesions [36]. There is a complex pathway of interactions taking place in the early endosome to sort the proteins to be recycled from those that are being degraded [37]. There are at least two distinct portions of the early endosome, a tubular compartment to which proteins to be recycled are targeted, and large vesicle like compartments, where proteins targeted for degradation are stored [38,39].

There are two types of endosomal recycling, slow recycling and fast recycling. Fast recycling occurs when the tubules in the recycling endosomes pinch off and are immediately reabsorbed into the plasma membrane [40,41]. The slow recycling may be where AmpA functions. During this process, the proteins are targeted to the endosomal recycling complex (ERC) [42]. There are two potential reasons for the slow versus fast recycling. The first is that the cell has tight regulation of the proteins on the plasma membrane. If the proteins are recycled too rapidly, it may negatively affect how the cell migrates or growth factor signaling may be over stimulated. But there is another, recently discovered cellular reason for proteins to enter the ERC. Some proteins need to go back through the Golgi via retrograde transport [43,44]. Once the proteins have gone back through the Golgi, they can now re-enter the secretory pathway.

AmpA is localized in what Charette suggests is an endosomal recycling complex in *Dictyostelium*[35]. AmpA also appears to be localized to a distinct portion of the ER and to the Golgi. However, Charette did not see any colocalization of p25 with calnexin or golvesin, a marker for the Golgi body [35]. Since AmpA does have some colocalization with the ER, it is possible that, after recycling, AmpA may be reprocessed through a portion of the ER and Golgi in order to be trafficked back to the plasma membrane.

By imaging live cells, it is seen that AmpA can be found on the extracellular surface. We have demonstrated that AmpA can be endocytosed, or recycled, because it can be extracellularly labeled in live cells with primary antibody and the antibody is then brought into the cell. Taken together these results seem to indicate a role for AmpA as a signaling molecule on the cell surface, possibly controlling adhesion and stimulating actin polymerization. Since AmpA is never detected free in the media in wild type cells yet can be detected on the cell surface in live cells, it is possible that it interacts with a membrane receptor protein as it passes through the ER, Golgi or secretory vesicles and is transiently presented on the extracellular face of the plasma membrane bound to its receptor. Possibly, AmpA functions on the cell surface to signal the down regulation of an adhesion protein by endocytosis. The presence of excessive AmpA functioning in this manner would result in a decrease in adhesion relative to wild type and the lack of AmpA could result in excess adhesion protein on the cell surface. However so far no such adhesion protein has been identified.

Zanchi et al. have used a temperature sensitive mutant of the secA gene to explore the relation between plasma membrane recycling and cell movement. The failure of exocytosis to take place in these mutants at the restrictive temperature results in a net uptake of plasma membrane which is suggested to restrict pseudopodial expansion [45]. It is possible that the role of AmpA in increasing endocytosis could alter plasma membrane recycling in the opposite direction resulting in increased pseudopod extension which could influence cell migration. However the *ampA* null cells do not show any alteration in endocytosis that we can reproducibly document so an explanation centering on general membrane turnover seems unlikely.

For cell migration it would appear that the effect of AmpA on substrate adhesion is more important than its role in actin polymerization, since *ampA* null cells can migrate as well as Wt cells under the right environmental conditions. Interestingly, wild type cells show far less variation in their migration rates as a result of environmental conditions (11.1 um/min on top of agar and 10.3 um/min under agar on glass) than either AmpA overexpressers (16.0 um/min on top of agar vs 10.2 um/min under agar on glass) or *ampA* null cells (5.0 um/min on top of agar vs 13.6 um/min under agar on glass). This suggests that there is an optimal level of AmpA that enables a cell to migrate consistently through a variety of environments and that too much or too little AmpA, while advantageous in some environments, is detrimental in others. Cells with an optimal amount of AmpA may not win the race on some surfaces but they can get to the bacteria and feed when faced with a wide variety of surfaces.

The *ampA* null cells clearly are more adhesive not only to the substrate during growth (Figure 3A) and development [19] but they are also more adhesive to each other [21]. When sitting at an air water interface on a cover slip the more adhesive *ampA* null cells have a much larger % of their surface area in contact with the substrate and the less adhesive AmpA overexpressing cells show a very reduced substrate contact area. This reverses when the cells are migrating under agar. The *ampA* null cells now show a reduction in substrate contact while the AmpA overexpressing cells show an increase. Interestingly the wild type cells show little difference in surface area contact under the two conditions (Figure 4B). It is possible that with their increased adhesion levels the *ampA* null cells adhere to the overlying agar as well as to the substrate, thereby spreading adhesion receptors over a greater portion of the cell surface and thus reducing the area of contact with the underlying substrate. The AmpA overexpressing cells may show more contact with the substrate under agar than they do at an air water interface not only because of the flattening effect of the agar but also because the agar layer on top of the cells may prevent aerial extension of the robust, overly actin rich pseudopods formed by these cells, directing them instead along the substrate and increasing the contact area. Another possibility suggested in a review by Lammerman and Sixt [46] is that while surface anchoring is essential for migration in a 2D environment it is possibly dispensable in a 3D environment where cells are closely surrounded by matrix materials. They base this suggestion on their studies in which genetic depletion of all 24 possible integrin heterodimers left unaltered the migration rate of neutrophils, dendritic cells and B cells in a 3D collagen gel. In this model the fact that the *ampA* null cells are overly adhesive may indeed restrict their motility in a soft 2D environment up top of agar but under agar this excess adhesion may not come into play. Likewise the advantage of the reduced adhesion of the AmpA overexpressing cells in a soft 2D environment may be lost in a 3D environment where dependence on adhesion receptors may be dispensable.

What is difficult to explain is the fact that AmpA both increases F-actin content and yet decreases adhesion and its absence has the opposite effect of increasing adhesion and decreasing F actin. This is the opposite of what would be expected since actin is a major component of cell adhesion. It is possible that AmpA acts as a signaling molecule on two different pathways and is required at a critical level to keep the pathways in balance. A better understanding of this will require a more extensive knowledge of the proteins that are involved in substrate adhesion during motility and their interaction with the actin cytoskeleton and the effects of membrane dynamics on their turnover. The results presented here suggest that AmpA is a player in these processes but its mechanism of action is unclear. AmpA likely functions as a signaling molecule binding to another protein or receptor or a complex of proteins. We have made many attempts to identify receptors or proteins that might interact with AmpA but the AmpA protein is 17% cysteine and has proved refractory to all affinity chromatography or pull down approaches for identifying an interacting protein. We have identified suppressors of AmpA overexpressing phenotypes and two of these have effects on endocytosis that influence cell migration but neither mutant identifies a candidate for an AmpA receptor or AmpA regulated adhesion protein [25,47]. It is possible that yeast 2 hybrid screens or identification of second site suppressors of *ampA* null phenotypes will eventually result in the identification of the partners with which AmpA interacts and allow for a definitive model for AmpA function.

## Conclusions

AmpA influences cell migration by influencing substrate adhesion and the area of cell substrate contact. Cells require an optimal level of Amp in order to migrate successfully over a wide variety of surfaces and environmental conditions. Excess AmpA on soft deformable surfaces like agar at an air water interface results in rapid migration but if the cells encounter a thick layer of bacteria they cannot generate the force to invade it even with the excess actin that they polymerize. This is presumably because of the decreased substrate adhesion. By contrast, the absence of AmpA in the null cells results in an almost complete failure of these cells to be able to migrate on top of agar at an air water interface and in a lawn of bacteria they jig and roll back and forth and can only make very small plaques. In a 3D environment under agar and on a hard surface like glass the advantage of excess AmpA is lost and the knockout cells that lack AmpA are able to migrate better than wild type cells possibly because of their increased adhesion or possibly because a 3D environment has a reduced requirement for adhesion [46]. Even though they have a reduced level of F-actin, it is sufficient to allow them to migrate better than wild type cells in this 3D environment.

AmpA is associated with an ER derived perinuclear compartment, Golgi and Golgi derived vesicles; it is present on the extracellular surface and is endocytosed and found in a perinuclear endocytic recycling compartment colocalized with p25, a protein used to identify a slow recycling compartment [35]. In spite of its effects on F-actin levels and cell migration AmpA is not associated with the actin cytoskeleton. Since AmpA does not have any transmembrane domains, only a hydrophobic leader sequence, it must require the partnership of another protein to be present on the cell surface. It is likely that as it transits through the ER and Golgi to the cell surface where it binds to a receptor. We postulate that this receptor plays a role in cell-cell and cell-substrate adhesion. AmpA could potentially control the life time of this receptor on the cell surface and in this way influence adhesion and possibly actin polymerization. But it is also possible that AmpA is a secreted autocrine ligand that signals through a surface receptor. Obviously these models rests on identification of an AmpA receptor or interacting protein which has so far not been identified. The SadA protein influences cell-substrate adhesion but is unlikely to be the AmpA receptor because SadA also influences phagocytosis and AmpA does not [14,15]. We have made many attempts to isolate this receptor but, with 17% cysteine in the protein, AmpA is very difficult to work with biochemically and none of the attempts to isolate interacting proteins have succeeded. We have used REMI mutagenesis to identify second site suppressors of AmpA overexpressing cell lines by selecting for reduced cell migration. Interestingly, all of these mutants influence cell migration and two out of three of these mutants influence or are associated with endocytic processes [25,47]. The best way to identify a potential AmpA receptor may be to use REMI mutagenesis to isolate second site suppressors of the *ampA* null mutant. Until a receptor for AmpA can be identified it will not be possible to further define how an optimal level of AmpA influences both cell substrate adhesion and actin polymerization to maintain a constant rate of migration over a wide variety of substrates.

## Methods

### Axenic Growth of *Dictyostelium*

Cell lines with the *ampA* gene knocked out or over expressed as well as methods for growing cells are described by [19]. For cell lines containing the blasticidin resistance cassette (bsr) or the G418 resistance cassette, 10ug/ml blasticidin S hydrochloride or 9.6 ug/ml G418 was included in the media respectively. Cells in late log phase (3-4 × 10^6^) were used in all experiments unless otherwise indicated. The *ampA* knockout and AmpA protein overexpressing cell lines are available from the Dictyostelium Stock Center (http://www.dictybase.org). An *ampA* null strain in which the blastocidin cassette has been removed by the lox-cre recombination system is described [25,48]. Cells were plated on LP agar plates (5 gm/liter Bactopeptone, 5 gm/liter Lactose, 2% agar) on a lawn of *E. coli* B/r for single colonies for plaque formation assays. The plates were incubated in a moist chamber at 22°C for 72 to 96 hours. In some experiments cells were plated on 1/2HL5 plates instead of LP agar plates. These richer plates allow for a thicker bacterial lawn. Plaques on LP plates were imaged after 5 to 6 days while plaques on 1/2HL5 plates were imaged after 11 days.

### Generation of mRFP-AmpA and AmpA-Tap tag fusion protein plasmids

#### AmpA-Tap tag plasmid

The *ampA* gene from the Eco RI site at the start of the promoter to the last amino acid codon (2.3Kb) was PCR amplified and cloned into the multiple cloning site between the Eco R1 and BamH1 sites of the pDDGal 16 vector [49]. The 5^′^ primer (Eco R1 site underlined) was 5^′^ CCGGAATTCTAAGAATATTATTATTATTATTA and the 3^′^ primer (Bam H1 site underlined) was 5^′^ CGCGGATCCTTGAGTTAAATTTTCACG. The beta-galactosidase sequence was removed by cutting with BamH1 and XhoI and replaced with the Tap tag sequence which was amplified from pBS1479 [31] using a 5’ primer containing a BamH1 site (underlined) (5^′^ AAGGGAACAAAAGCTGGAGGATCCATG) and a 3^′^ primer containing an XhoI site (underlined) 5^′^ CTGACGCTCGAGTTAGGTTGACTTCCCCGCGGA to obtain a plasmid called pKL1. The pKL1 plasmid has a KpnI site immediately upstream of the EcoR1 site at the start of the AmpA promoter. The AmpA 3^′^ downstream region from the AmpA termination codon to a site ~1000 base pairs downstream was PCR amplified. The 5^′^ primer containing a Bam H1 site (underlined) was 5^′^ AAGGGAACAAAAGCTGGAGGATCCATG and the 3^′^ primer containing a Not I site (underlined) was 5^′^ TCAAGGATGAGCGGC CGCAATTCTCTATGGTCAACATTA. This PCR fragment was ligated into pLPBLP [48] at the BamH1 Not1 sites. This plasmid which contains the floxed blasticidin cassette was called pKL2. The 215 bp *ampA* terminator site was PCR amplified from the full length genomic clone of *ampA* in pGem3 using a 5^′^ primer containing an Xho I site (underlined) 5^′^ GGTTGTTGCCCATCTCGAGAAAATTTAACTCAA and a 3^′^ primer containing a Hind III site (underlined) 5^′^ GCGGCCAAGCTTTTAATAGTGTGTTATTA. Instead of simply adding the restriction sites onto the 5^′^ end of each primer, two bases were changed in the *ampA* sequence to create the sense primer and one base was changed to create the antisense primer. This was done because it is hard to find segments with a high enough GC content for PCR. Therefore, two short primer binding sites with a relatively high GC content were chosen that flanked the *ampA* terminator region at the 3^′^ end of the gene. In the sense 5^′^ primer, GTCGTG was changed to CTCGAG to create an XhoI site, while in the antisense 3^′^ primer AAGTTT was changed to AAGCTT to create a HindIII site. This PCR fragment was cloned into the pBluescript II vector at the Hind III –XhoI site to obtain plasmid pKL3. The *ampA* promoter, coding region, and in frame fusion to the Tap tag was excised from pKL1 by Kpn I – XhoI and subcloned into the pKL3 plasmid at the KpnI-XhoI site so that the *ampA* terminator sequence was immediately downstream of the AmpA-Tap tag fusion protein gene. This generated plasmid pKL4. The KpnI-Hind III fragment from pKL4 was then subcloned into the KpnI –Hind III site of pKL3 to place the *ampA*-Tap tag –Terminator sequence adjacent to the blastocidin cassette and 3^′^ downstream *ampA* sequence in the AmpA-Tap tag vector shown in Additional file 5.

The mRFP-AmpA plasmid was constructed so that the AmpA hydrophobic leader sequence (MLNKLILLLILSSCLVLSVKSEV – predicted cleavage site underlined) preceded the mRFP coding sequence [32] which was followed by the remainder of the *ampA* coding sequence starting with the amino acid “N” which immediately follows the hydrophobic leader. A PCR copy of the *ampA* coding sequence starting at the amino acid (N) immediately following the hydrophobic leader and continuing 193 nucleotides downstream past the unique Age I restriction enzyme site in the *ampA* coding sequence was generated. The 5^′^ primer containing a BamH1 site (underlined) was 5^′^ GCGCGGATCCAATGTTGATTGCTCCCTCG and the 3^′^ primer containing a ClaI site (underlined) was 5^′^ CGCGATCGATGGTTGGTGGGAGAGTACATGGA. This PCR fragment was cloned into the 339–3 mRFPmars-BsrH plasmid [32] to generate an in frame fusion of mRFP-mars to AmpA coding sequence distal to the hydrophobic leader (mRFP-mars-AmpA-C-terminal). This 880 base pair DNA fragment was excised from the BsrH plasmid as a HindIII-ClaI fragment and subcloned into the HindIII –ClaI site of a pBluescript plasmid (Stratagene) to generate pBlue1. A 1550 base pair PCR fragment was generated that included the 5^′^ AmpA promoter sequence starting upstream of the unique Bgl II site and ending at the last amino acid of the AmpA hydrophobic leader sequence (V). The 5^′^ primer for this PCR product containing an Eco RI site (underlined) was 5^′^ CGCGGAATTCACAACTAATTGTAATACCTGCAATTG and the 3^′^ primer containing a Hind III site (underlined) was 5^′^ GCGCAAGCTTAACTTCACTTTTAACTGATAGTACC. This fragment was cloned into the pBlue1 plasmid at the Eco RI –Hind III site to generate mRFP-AmpA-pBlue2. Next, the pBlue2 plasmid was cut with BglII and AgeI. These are unique restriction enzyme sites in the *ampA* promoter and coding region respectively. Cutting at these sites excises the mRFP-AmpA construct from the pBlue2 vector. This BglII-AgeI fragment was cloned into a full length *ampA* Eco RI genomic DNA fragment in a pGem3 vector and replaced the endogeneous BglII-AgeI DNA fragment with the BglII-AgeI DNA fragment that now contained the AmpA hydrophobic leader fused in frame to the N-terminus of the mRFPmars coding sequence which is fused in frame N-terminal to the remainder of the AmpA coding sequence. Finally the floxed blastocidin cassette from the PLPBLP plasmid [48] was excised with PstI and XmaI and cloned into the PstI-XmaI restriction sites in the multiple cloning site of the pGem3-mRFP-AmpA vector (Additional file 6).

#### Transformation of *Dictyostelium*

Plasmids containing GFP fused to ABD120 [28], Calnexin [33] or N-Golvesin [34] were acquired from the *Dictyostelium* Stock Center. All plasmids were inserted into the *Dictyostelium* cells via electroporation [50].

#### Live Cell Microscopy of Transformed Strains

Cells were placed on chambered coverslips overnight in HL5 media for 16–18 hours and imaged on a Leica SP5 confocal microscope. For time courses, images were acquired every 5–10 seconds for 5 minutes. All data were analyzed using either the Volocity Program (Perkin Elmer) or Image J (NIH).

#### Endocytosis

Wild type Ax3, *ampA* knock out, and AmpA overexpressing cells were grown to a density of approximately 3 × 10^6^ cells/ml and assayed as in [51] with the exception that the media was supplemented with 2 mg/ml FITC dextran without centrifugation of the cells. One milliliter aliquots were taken at times 0, 20, 40, 60 and 120 minutes. Fluorescence was read on a spectrofluorimeter (Bio-Rad) using a 488nm excitation filter and a 520nm emission filter. The fluorescence was compared against a standard curve and the micrograms of FITC dextran that were endocytosed per 1 × 10^6^ cells were determined.

#### Exocytosis

For exocytosis assays, the cells were grown to a density of 3 × 10^6^ cells/ml. The growth medium was supplemented with 2 mg/ml FITC dextran and the cells were incubated for 3 hours. The cells were then centrifuged and the media was replaced with unsupplemented HL5. One milliliter aliquots were taken at 0, 20, 40, 60, and 120 minutes. The aliquots were washed once with HL5 and once with endocytosis wash buffer. The procedure then was as described above [51].

#### Phagocytosis

Phagocytosis was assayed by following the uptake of carboxylated fluorescent latex beads (FITC#15702) from PolyScience, Warrenton Pa. (1 um in diameter) as described [52]. Fluorescence was determined using a BioRad spectrofluorimeter.

#### FM-64 staining

FM-64 Staining is described in [53]. Cells were imaged using a Leica SP5 confocal microscope.

#### Phalloidin binding

Phalloidin binding to quantify the amount of polymerized actin was carried out according to [54]. Cells were grown to mid-log phase and 3 × 10^7^ cells were centrifuged and the pellet resuspended in 1 ml 20 mM K_2_KPO_4_. A 100 ul sample of cells was placed in 1 ml fixing solution (3.7% formaldehyde, 10 mM PIPES, 0.1% TritonX-100, 20 mM K_2_KPO_4_, 5 mM EGTA, 2 mM MgCl_2_ and 250 nM Alexa fluor 488-phalloidin (Molecular Probes) and incubated at room temperature for 1 hour. Cells were then centrifuged for 5 minutes at 2000 rpm in a microcentrifuge and after one wash in 20 mM K_2_KPO_4_ the pellet was resuspended in 1ml methanol and vortexed briefly. Cell debris was removed by a brief centrifugation in a microfuge and the FITC-fluorescence in the supernatant was determined using a BioRad versafluor fluorometer with a 488 nm excitation filter and a 520 nm emission filter. Data points were taken in triplicates and the assays repeated 3 times on different days with different batches of cells. Nanomoles of phalloidin were determined by comparison to a known concentration standard curve assayed in parallel.

#### Cell adhesion assay

2 × 10^6^ cells in 10 ml were placed on 60x15 mm plates (Sarstedt) and allowed to incubate overnight in a humid chamber. The following day, the cells were placed on a shaking platform at the indicated shaker speeds (rotations per minute, RPMs) for 45 minutes. The supernatant was then removed and the number of cells released from the plates was determined. Medium (10 ml) was then re-added to the plates which were scraped and the number of cells that had remained attached to the plate was assayed. The number of cells that remained attached was calculated as a percentage of total cells.

#### Reflection imaging to determine the percent of the cell surface in contact with the substrate

Cells were allowed to adhere in chambered cover slips overnight. The following day they were imaged on the SP5 Confocal microscope in both transmitted light mode and reflection mode. The argon laser was used and the excitation wavelength was 488 nm for reflection imaging. This allowed the visualization of the area of the cell in direct contact with the substrate. The images were imported into ImageJ and the ratio of the area of the cell in contact with the substrate to the total area of the cell was determined.

#### Migration of growing cells on top of and under agar

Migration of cells under agar was according to [30] using 0.8% agar. The agar was formed either on chambered glass cover slips or on plastic dishes. Cells in HL5 were placed into wells cut in the agar. Folic acid (0.5 mM) was added to a trough 5 mm away from the trough containing the cells 1 hour prior to the addition of cells to allow a gradient to form. Cells were imaged from underneath using a 40x objective on a Leica SP5 scanning confocal microscope. Imaging was initiated 3 to 5 hours after the addition of cells to the wells. Images were collected at 10 second intervals for periods of 5 min.

Migration of cells on top of 1% agar in 20 mM NaKPO4 buffer was as described by [26] except that a very thin layer of agar was spread on a chambered cover slip. Cells were grown to mid log phase in axenic culture. Cells were centrifuged and resuspended at a volume of 1 × 10^7^ cells/ml. Ten microliters of cells were spotted 1 mm away from a 5 ul drop of 0.5 mM folic acid. The folic acid was spotted on the agar 1 hr before the cells to allow diffusion of the folic acid through the agar. Imaging was started 4 hours after the addition of cells. Cells were imaged from underneath with a 40x objective equipped with a correction collar on an inverted Leica SP5 confocal microscope. Because the thickness of the thin layer of agar varies from dish to dish it was necessary to use the zoom function to get the very clearest images of cells on top of the agar. This means that the magnification is not always identical from image to image in these experiments but scale bars are imbedded in each image. Images were captured every 10 seconds for 5 min intervals. Image analysis was done using the Image J (NIH), Volocity (Perkin Elmer), Metamorph (Universal Imaging) and DIAS software (Solltec). Each image or video series was calibrated with its own scale bar.

#### Analysis of cell motility

Directionality is a measurement of how well cells move in a single direction and is determined by the ratio of productive distance to total distance. Total distance is the sum of how far the cell traveled. The productive distance is how far the cell traveled from the point of origin over the time of the measurement and is determined by the length of a straight line from the starting position to the ending position (Euclidian distance). The chemotactic index is used to determine how well cells migrate to a source of chemoattractant and is determined by taking the cosine of the angle between a line that parallels the gradient and a line created between the cell’s end point and the cell’s starting point. A value of 1 indicates the cell is moving directly up the gradient and a value of −1 indicates a cell is moving directly against the gradient. Roundness is the ratio of the length to the width of the cell converted to a percent. 100% is totally round.

#### Western Blotting

Strains were grown to log phase. 1 × 10^6^ cells were harvested by centrifugation and the pellet was resuspended in 30ul Laemmli sample buffer [55]. Polyacrylamide gel electophoresis (PAGE) was performed under the standard conditions [55]. For Western blots the proteins were transferred onto nitrocellulose using a Bio-Rad transfer apparatus. The transfer buffer was continuous buffer (0.292% glycine, 0.58% Tris, 0.0376% SDS, 20% MeOH). The nitrocellulose was blocked overnight with 2.5% non-fat dry milk in TBST (10 mM Tris–HCl 150 mM NaCl, 0.05% Tween 20). The blot was then probed with either the anti-actin antibody (1:1000 dilution 224-236-1 Hybridoma Bank) [56] or the anti-TAP antibody (1:1000, Open Biosystems). Initial lots of anti-Tap antibody showed no cross reactivity with any *Dictyostelium* proteins and were suitable for immunofluorescence imaging but subsequent lots contained cross reacting antibodies and were only suitable for western blots. Secondary antibodies were goat anti-mouse conjugated to alkaline phosphatase from Jackson Immunoresearch.

#### Immunofluorescence

Cells were grown to a high density (6 × 10^6^ cells/ml). 100ul were spotted onto a cover slip that had been placed in a petri dish with moistened paper towels. The cells were allowed to adhere to the cover slips for 18 hours. The medium was removed and the cells were fixed with 4% formaldehyde in 20 mM Na_2_KPO_4_ buffer for 15 minutes at room temperature. The cells were permeabilized in anhydrous MeOH with 1% formaldehyde at −20°C for five minutes. The cells were incubated with anti-tap (Open Biosystems) or anti-mRFP (Chromtek) antibodies for 2 hours followed by incubation for 2 hours with secondary antibodies (Invitrogen). The cover slips were washed 3x in PBS and mounted on microscope slides (Corning) coated with Prolong Gold Antifade Reagaent (Molecular Probes). For actin staining, cells were fixed in 0.3% gluteraldehyde and permeabilized with 0.1% TritonX-100 in PBS (pH 7.4) buffer. Where utilized, primary antibodies were applied followed by incubation with secondary antibodies. Alexa fluor 488-phalloidin (Molecular Probes) at a 1:500 dilution was included in the secondary incubation to label F-actin. Alexa fluor-594 conjugated deoxyribonuclease I (Molecular Probes) was added along with the phalloidin to label unpolymerized G-actin.

For localization of mRFP-AmpA or AmpA-Tap tag to the plasma membrane, cells were incubated for 10 minutes at 4^o^ with the DiI membrane stain (Invitrogen) resuspended per the manufacturer's instructions. The cells were then incubated for 1 hour at room temperature or at 4°C with rat anti-RFP primary antibody (Chromtek) or rabbit anti-tap antibody (Open Biosystems). They were washed 3x with HL5 and then incubated in secondary goat anti-rat or goat anti-rabbit antibodies conjugated to FITC. They were again washed with HL5 and then fixed with 3.7% formaldehyde in 20 mM Na_2_KPO_4_ buffer. They were washed and mounted on cover slips using Prolong Gold Antifade (Invitrogen).

Imaging of fixed cells was done on a Leica SP confocal microscope. For FITC and Alexa fluor 488-conjugated antibodies the Argon laser line 488 was used for excitation (20%) and an emission band width of 500-550 nm was used. For 594 Alexa conjugated antibody imaging excitation was with the DPSS laser line 561 (15%) and HeNe laser line 594 (30%) and emission was detected at a range of 600 nm-767 nm. In DiI staining the DPSS laser line 561 nm (30%) was used for excitation and an emission range of 604-767 nm was used. For DAPI staining and nuclei visualization, the stain was excited with the Diode laser line 405 nm (8%) and emission was recorded at 430-477nm.

#### Quantitation of fluorescent images of F-actin staining in cells

Imaging of fluorescently labeled cells was done in pairs of Wt and *ampA* nulls or AmpA overexpresser and Wt. In each pair the gain and laser power on the confocal microscope was set so that the most intense pixels of the pair (Wild type or Overexpresser) were set equal to 255 grey scale units so that the pair of fluorescent images would be in the linear range (0 to 255 grey scale values) and could be quantified relative to each other. Z series were collected and converted to an extended focus 3D reconstruction and a 2D image of the 3D rendering was quantified in image J. The integrated pixel intensity per cell area in um^2^ was calculated.

#### Microscopy and Image Analysis

*Dictyostelium* plaque formation on bacterial lawns was viewed under an Olympus dissecting scope. All plaque images were acquired at a magnification of 22x. For high magnification viewing of cells within plaques, cells were imaged from underneath the petri dish using a Leica DM IRB microscope with a 40x objective with a correction collar. Images were collected using a DC330 video camera (DAGE-MTI, Inc., Michigan City, IN, USA) and a frame grabber. Images were digitized, processed, and analyzed using the Metamorph image processing system, (Universal Imaging, West Chester, PA, USA). Timelapse videos of cells migrating in plaques were taken at 1 to 2 minute intervals for 30 to 60 min. The microscope was refocused after collection of each image in the series. Cell migration was tracked and the velocity determined using the centroid-tracking program in the Metamorph image processing software. To track individual cells at the periphery of plaques, each image in the video stack was enlarged 400x to clearly see individual cell outlines.

## Competing interests

The authors declare that they have no competing interests.

## Authors’ contributions

EFN and CLP designed and carried out cell migration experiments. EFN designed and carried out live and fixed cell imaging and reflection microscopy experiments. CLP and HNC designed and assayed migration in bacterial lawns. HNC generated and characterized the AmpA overexpressing strain. JSK carried out substrate adhesion experiments and provided helpful insight to the direction of the studies. NB helped to maintain and construct the cell lines and preformed the cell fractionations and western blots. EFN helped to draft the manuscript and helped to plan the studies. DDB designed the studies, conceived of the project and helped draft the manuscript and was responsible for obtaining funding. All authors have read and approved the manuscript.

## Authors’ information

DDB is associate professor at the University of Maryland, Baltimore County. This work constituted partial completion of a Ph.D. thesis for EFN, JSK and HNC and an M.S. thesis for CLP. CLP is currently the manager of the Keith R. Porter Light and Electron Microscopy Facility at University of Maryland, Baltimore County. NB was an undergraduate research intern and a Meyerhoff Scholar at University of Maryland, Baltimore County.

## Supplementary Material

Additional file 1 **Phagocytosis is not significantly altered in*ampA*mutants.** Supplemental figure and legend. Click here for file

Additional file 2 Quick Time Movie Wt cells migrating on top of agar to folic acid −2 frames per second. Click here for file

Additional file 3 Quick Time Movie KO cells migrating on top of agar to folic acid-2 frames per second. Click here for file

Additional file 4 Quick Time Movie OE cells migrating on top of agar to folic acid-2 frames per second. Click here for file

Additional file 5 ***Amp *A influences the level of F-actin in growing *Dictyostelium *cells.** Supplemental figure and legend. Click here for file

Additional file 6 AmpA overexpressing cells cannot penetrate a thick bacterial lawn. Click here for file

Additional file 7 The area of the cell in contact with the substrate is influenced by AmpA in an environment dependent manner. Click here for file

Additional file 8 Quick Time Movie Wt cells migrating under agar to folic acid-2 frames per second. Click here for file

Additional file 9 Quick Time Movie KO cells migrating under agar to folic acid-2 frames per second. Click here for file

Additional file 10 Quick Time Movie OE cells migrating under agar to folic acid-2 frames per second. Click here for file

Additional file 11 **An AmpA-Tap tag fusion protein vector introduced into cells as a linear KpnI-Not I DNA fragment expresses the AmpA-tap tag fusion protein and retains a wild type phenotype while the same plasmid introduced as a covalently closed circular bacterial plasmid shows an AmpA over expressing phenotype.** Supplemental figure and legend. Click here for file

Additional file 12 **A mRFP-AmpA fusion protein plasmid introduced as an extrachromosomal plasmid correctly expresses the mRFP-AmpA fusion protein and has an overexpressor phenotype.** Supplemental figure and legend. Click here for file

Additional file 13 **AmpA Tap tag fusion proteins show the same distribution of AmpA protein in vesicles throughout the cells and in a perinuclear compartment as the mRFP-AmpA tagged construct. AmpA-tap tagged OE and AmpA-tap tagged Wt strains show a similar distribution of AmpA protein within the cell.** Supplemental figure and legend. Click here for file

Additional file 14 **Overexpression of *ampA* causes multiple endocytic cups to form repeatedly at the same site.** Supplemental figure and legend. Click here for file

Additional file 15 **Quick Time Movie; Wt cells carrying the ABD-GFP plasmid (Bsr) undergoing endocytosis.** Movie-2 frames per second. Click here for file

Additional file 16 **Quick Time Movie; AmpA overexpressing cells carrying the ABD-GFP plasmid (Bsr) undergoing endocytosis.** Movie-2 frames per second. Click here for file

Additional file 17 **Quick Time Movie; AmpA overexpressing cells carrying the ABD-GFP plasmid (Bsr) undergoing endocytosis.** Movie-2 frames per second. Click here for file

Additional file 18 **Endosomes acidify properly in *ampA *mutants.** Supplemental figure and legend. Click here for file

Additional file 19 **There is no defect in the formation of contractile vacuoles in *ampA *mutants.** Supplemental figure and legend. Click here for file
