# Supplementary material for: The adhesion modulation protein, AmpA localizes to an endocytic compartment and influences substrate adhesion, actin polymerization and endocytosis in vegetative Dictyostelium cells
Source: BMC Cell Biol. 2012 Nov 5;13:29. doi: 10.1186/1471-2121-13-29 (PMC3586950; doi:10.1186/1471-2121-13-29)
Supplement: Additional file 1 — Phagocytosis is not significantly altered inampAmutants. Supplemental figure and legend. [file 1471-2121-13-29-S1.pdf]

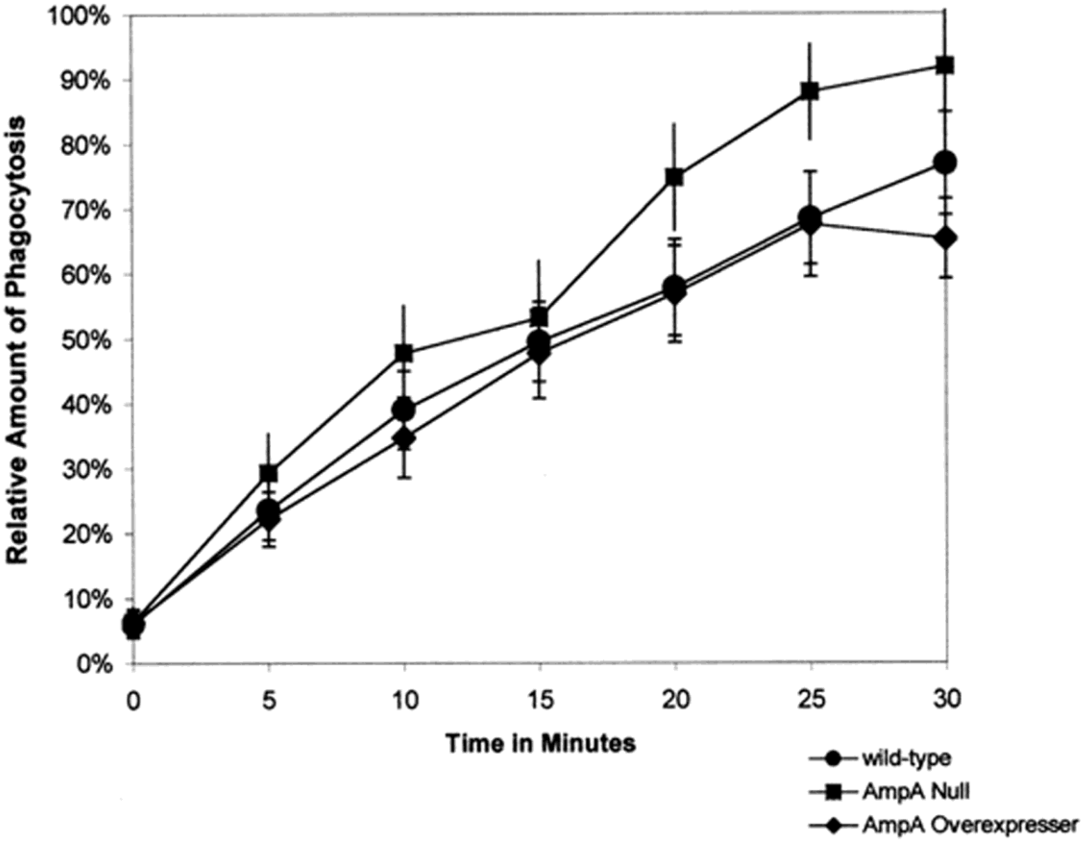

Additional File 1 Phagocytosis is not significantly altered in *ampA* mutants
Phagocytosis was measured by the uptake of latex beads labeled with FITC. The amount of phagocytosis was determined by the amount of fluorescence the cell ingested compared to the amount available. 100 percent relative phagocytosis is equal to complete ingestion of all beads in the solution. The results are the average of 3 repeats for each strain. The error bars are the standard error of the mean.
