# Supplementary material for: The adhesion modulation protein, AmpA localizes to an endocytic compartment and influences substrate adhesion, actin polymerization and endocytosis in vegetative Dictyostelium cells
Source: BMC Cell Biol. 2012 Nov 5;13:29. doi: 10.1186/1471-2121-13-29 (PMC3586950; doi:10.1186/1471-2121-13-29)
Supplement: Additional file 5 — Amp A influences the level of F-actin in growing Dictyostelium cells. Supplemental figure and legend. [file 1471-2121-13-29-S5.pdf]

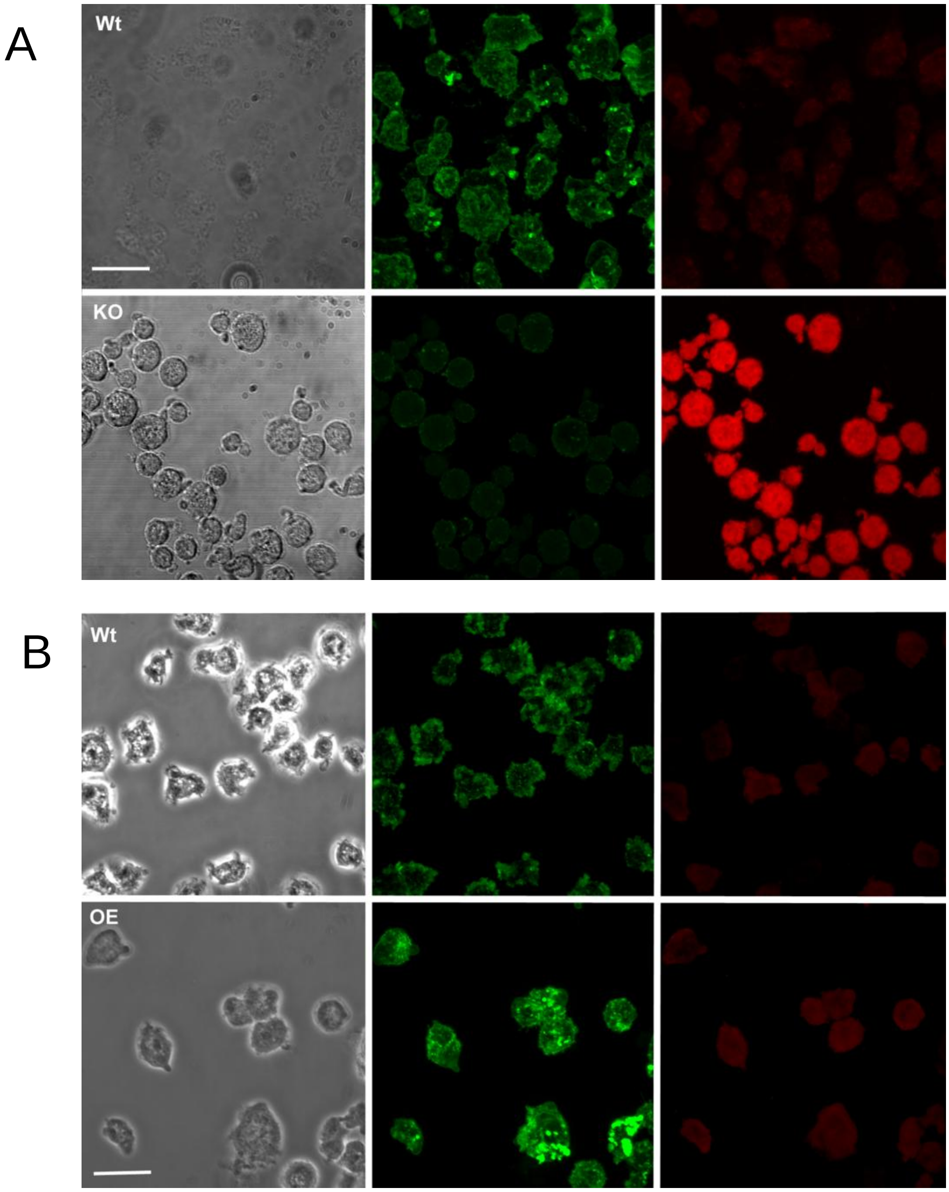

Additional File 5 *amp*A influences the level of F-actin in growing *Dictyostelium* cells. Fields of Wt and ampA knockout cells (**A)** or Wt and ampA overexpressing cells (**B**) were imaged in parallel with the laser and gain settings optimized for the most intensely staining cell line of the pair so that the amount of signal would be in a linear grey scale range. Cells grown to 4x10e6 cells per ml were deposited on coverslips for 1 hour prior to staining for F-actin with Alexa-488 phalloidin (green) and TRITC-DNAse I to detect G actin (red). Transmitted images are in the left panel. Images were collected using a 100x oil 1.4 N.A. objective. A 3D reconstruction from a z series is shown. Scale bar is 20um.
