# Supplementary material for: The adhesion modulation protein, AmpA localizes to an endocytic compartment and influences substrate adhesion, actin polymerization and endocytosis in vegetative Dictyostelium cells
Source: BMC Cell Biol. 2012 Nov 5;13:29. doi: 10.1186/1471-2121-13-29 (PMC3586950; doi:10.1186/1471-2121-13-29)
Supplement: Additional file 6 — AmpA overexpressing cells cannot penetrate a thick bacterial lawn. [file 1471-2121-13-29-S6.pdf]

A Migration of Cells Out of Plaques on Rich Broth Plates

	Total Distance um in 30 min	Productive Distance um in 30 min	Directionality	Velocity um/min
Wt	9.11+/-0.6	6.9 +/-0.3	0.75	0.3 +/-0.02
KO	10.1 +/- 0.4	4.4 +/- 0.2 *	0.4*	0.5 +/- 0.03 *
OE	17.5 +/- 0.8 *	11.9 +/- 0.7 *	0.7	0.6 +/- 0.03 *

* indicate significant deviation from the wild type control, p value <0.05

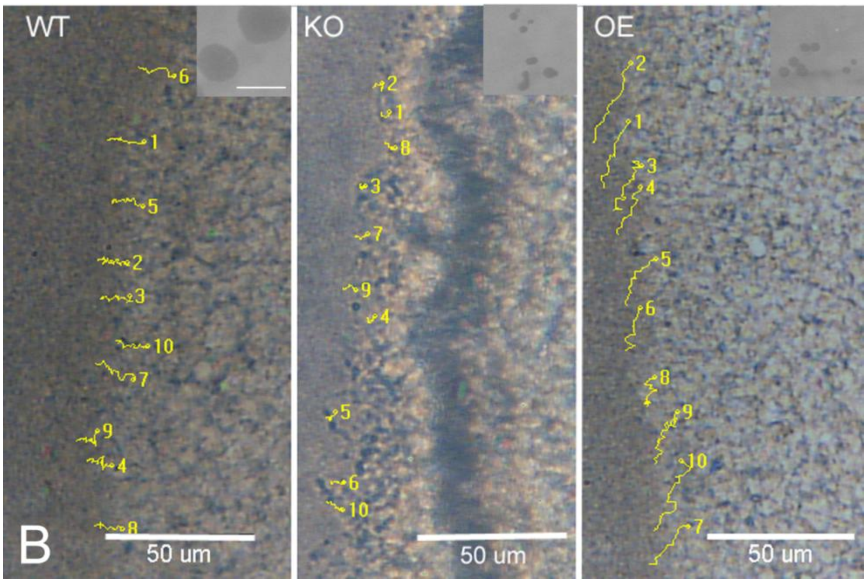

Additional File 6. <u>AmpA overexpressing cells cannot penetrate a thick bacterial lawn.</u>
A) Quantification of cell migration out of the plaques shown in B. The data are the averages +/- the standard error of the mean. * indicate significant deviation from the wild type control ($P < 0.05$). Data are the compilation of 30 to 60 cells tracked in 3 different experiments. Productive distance is the Euclidian distance a cell traveled from the start of imaging to its furthest point into the bacterial lawn. Directionality is the productive distance traveled divided by the total distance traveled and is a measure of how progressively or how straight a cell migrates. **B)** On rich broth plates AmpA overexpressing cells migrate rapidly but are unable to penetrate the denser bacterial lawn and instead migrate around the plaque circumference. Cells at the periphery of plaques formed on lawns of bacteria were imaged from underneath the petri dish with a 40x objective with correction collar. From left to right; wild type, *ampA* null (KO) and AmpA overexpressing cells (OE). For tracking centroids of individual cells, images were magnified 400X to see outlines of individual cells. The centroids of the migrating cells at the periphery of each of the plaques shown were tracked at 1minute intervals for 30 minutes. The yellow lines depict the path followed by each cell. The cells are moving from right (inside the plaque) to left (into the bacterial lawn) Scale bars are 50um. Insets in B are photomicrographs of plaques formed on high density bacterial lawns by wild type, *ampA* null and AmpA overexpressing cells 96 hr after plating. Scale bars in the insets are 1000 um.
