# Supplementary material for: The adhesion modulation protein, AmpA localizes to an endocytic compartment and influences substrate adhesion, actin polymerization and endocytosis in vegetative Dictyostelium cells
Source: BMC Cell Biol. 2012 Nov 5;13:29. doi: 10.1186/1471-2121-13-29 (PMC3586950; doi:10.1186/1471-2121-13-29)
Supplement: Additional file 7 — The area of the cell in contact with the substrate is influenced by AmpA in an environment dependent manner. [file 1471-2121-13-29-S7.pdf]

A

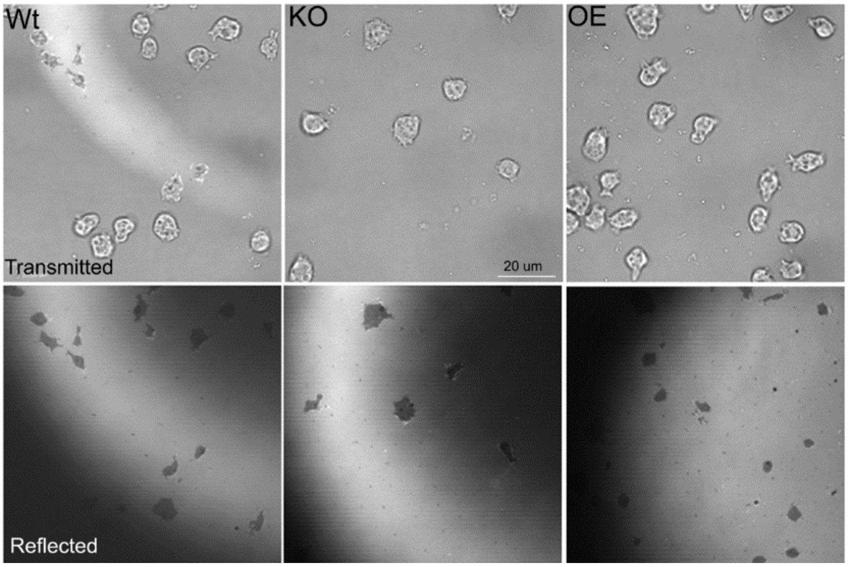

B

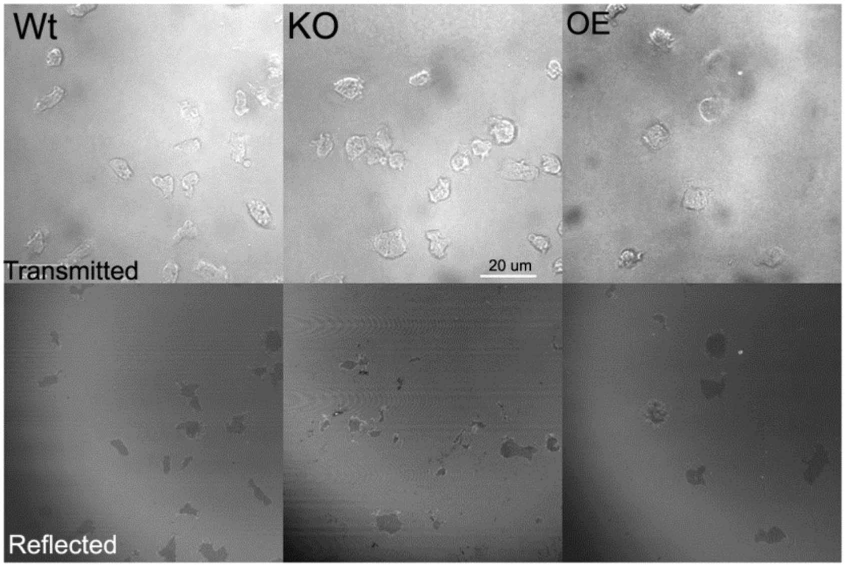

Additional File 7 The area of the cell in contact with the substrate is influenced by AmpA in an environment dependent manner.

A) AmpA OE cells sitting on glass cover slips have less contact with the substrate than Wt cells and *ampA* null cells have more: Cells were incubated overnight in chambered cover slips and imaged under transmitted light (top) and in reflection mode (bottom) on an SP5 confocal microscope. The dark areas in reflection mode indicate were the cells are in contact with the substrate. Scale bar is 20μm. **B)** Migration on glass under agar alters the cell area in contact with the substrate; overexpressing cell have more contact and knockout have less: Cells were allowed to migrate under agar on glass cover slips for 3 to 4 hours and then imaged under transmitted light (top) and in reflection mode (bottom) as in A. Scale bar is 20um. Quantification is shown in Figure 3B
