# Supplementary material for: The adhesion modulation protein, AmpA localizes to an endocytic compartment and influences substrate adhesion, actin polymerization and endocytosis in vegetative Dictyostelium cells
Source: BMC Cell Biol. 2012 Nov 5;13:29. doi: 10.1186/1471-2121-13-29 (PMC3586950; doi:10.1186/1471-2121-13-29)
Supplement: Additional file 11 — An AmpA-Tap tag fusion protein vector introduced into cells as a linear KpnI-Not I DNA fragment expresses the AmpA-tap tag fusion protein and retains a wild type phenotype while the same plasmid introduced as a covalently closed circular bacterial plasmid shows an AmpA over expressing phenotype. Supplemental figure and legend. [file 1471-2121-13-29-S11.pdf]

A

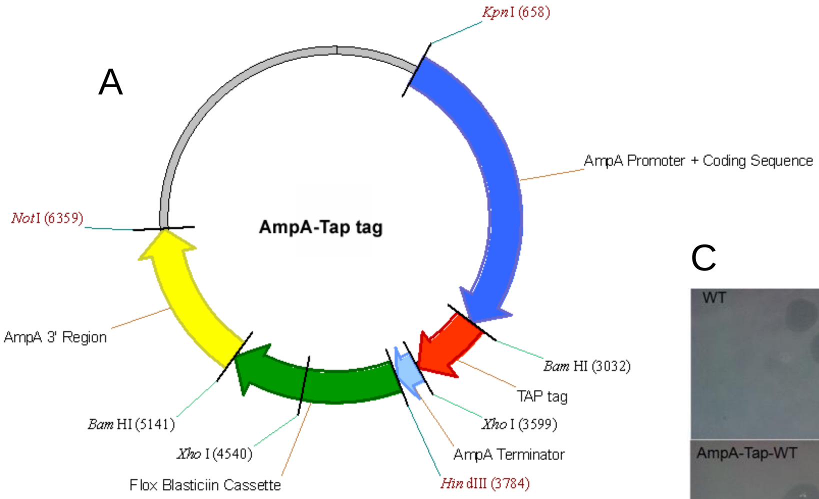

B

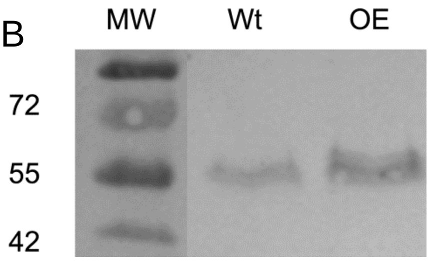

C

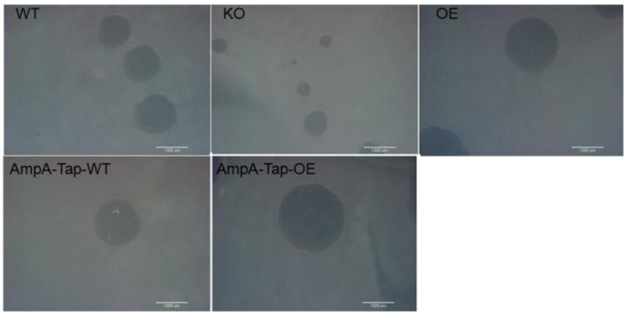

D

Strains	Plaque Sizes (mm^2)
Wt	1.41 +/-0. 17
KO	0. 28 +/- 0.04*
OE	2.33 +/- 0.28*
AmpA-Tap-Wt	1.90 +/- 0.23
AmpA-Tap-OE	3.168 +/-0. 29*

E

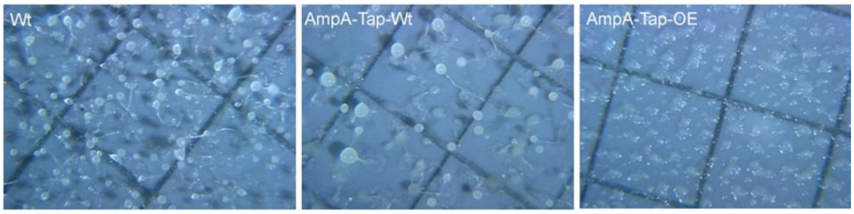

Additional File 11. AmpA-Tap tag fusion protein vector introduced into cells as a linear KpnI-Not I DNA fragment expresses the AmpA-tap tag fusion protein and retains a wild type phenotype. The entire plasmid introduced as a circular DNA has an AmpA overexpressing phenotype. **A)** The AmpA-Tap tag fusion vector was constructed with the *amp*A promoter and full coding sequence followed in frame by the Tap tag, the *ampA* terminator sequence, a floxed blastocidin cassette and1,000 base pairs of 3' non coding sequence flanking the *ampA* gene. The entire plasmid introduced into Wt cells as a closed circular DNA produced an AmpA overexpression phenotype (AmpA-Tap tag-OE). The linearized KpnI-NotI DNA fragment containing the AmpA-Tap tag fusion protein, blastocidin resistance cassette and 3'region introduced into Wt cells gives a Wt phenotype (AmpA-Tap tag-Wt). **B)** Western blot of AmpA-Tap tag protein from $1x10^6$ AmpA-Tap tag-Wt and AmpA-Tap tag-OE cells probed with anti tap tag antibody. The AmpA tap tag protein is the expected 55Kd molecular weight. Quantification indicates that the Amp Tap tag OE cells make 3x more AmpA-Tap tag protein than the AmpA-Tap tag–Wt cells. **C**) Wt, *ampA* null and AmpA overexpressing cells plus the AmpA-Tap tag, Wt and OE were plated on bacterial lawns for 4 days and the plates were photographed. Scale bars 1000um. **D)** The areas of the plaques were measured using the Metamorph object measuring program. Average plaque area +/- the standard error of the mean is recorded Significance was determined by a two tailed students T test. * indicates significant difference from Wt with a P value less than 0.05. Over 35 plaques were measured for each cell line. The AmpA-Tap tag Wt strain has a plaque size not significantly different from wild type. AmpA-Tap tag-OE strain has a plaque size larger than AmpAOE. **E)** Wt, AmpA Tap-tag Wt and AmpA-Tap tag-OE cells plated for development for 24 hours. Wt and AmpA-Tap tag- Wt have advanced to the culminant stage while the AmpA-Tap tag-OE strain has arrested at mound stage, characteristic of the AmpA overexpressing cell lines (Varney et al 2006a).
