# Supplementary material for: The adhesion modulation protein, AmpA localizes to an endocytic compartment and influences substrate adhesion, actin polymerization and endocytosis in vegetative Dictyostelium cells
Source: BMC Cell Biol. 2012 Nov 5;13:29. doi: 10.1186/1471-2121-13-29 (PMC3586950; doi:10.1186/1471-2121-13-29)
Supplement: Additional file 12 — A mRFP-AmpA fusion protein plasmid introduced as an extrachromosomal plasmid correctly expresses the mRFP-AmpA fusion protein and has an overexpressor phenotype. Supplemental figure and legend. [file 1471-2121-13-29-S12.pdf]

A

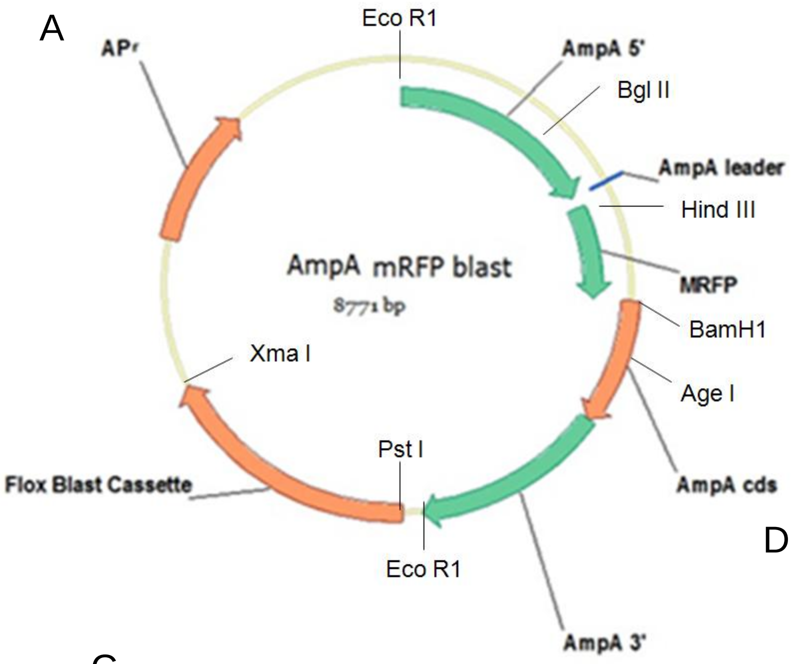

B

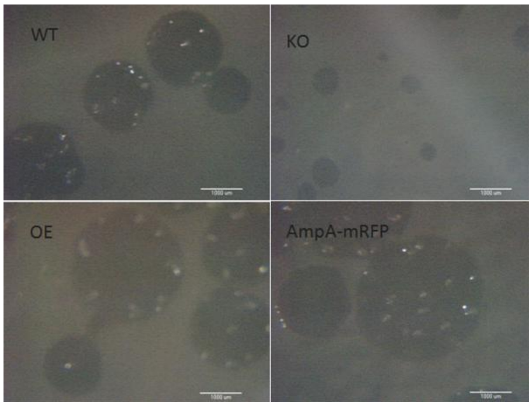

C

Strains	Plaque sizes (mm^2)
Wt	2.64 +/- 0.40
KO	0.43 +/- 0.063*
OE	5.42 +/-0.52*
mRFP-AmpA	6.27 +/- 0.56*

D

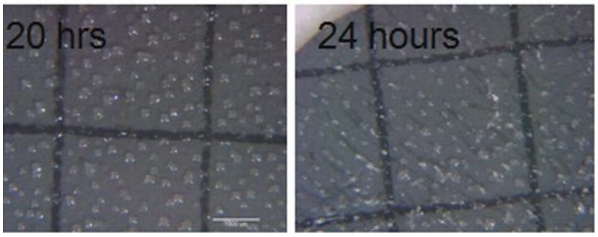

E

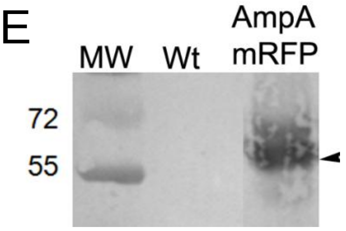

Additional File 12 mRFP-AmpA fusion protein introduced on a circular DNA plasmid correctly expresses the mRFP-AmpA fusion protein and has an overexpression phenotype. **A)** Plasmid containing the mRFP-AmpA fusion protein. The entire promoter and AmpA coding sequence up to the codon for the last amino acid of the hydrophobic leader sequence is fused in frame to mRFP. After the last mRFP codon, the AmpA coding sequence is cloned in frame starting with the codon for the amino acid that follows the last amino acid of the hydrophobic leader through the ampA terminator site and 1000 base pairs of sequence down stream of ampA. This places the AmpA hydrophobic leader and proteolytic cleavage site in front of the mRFP coding region and the remainder of the *ampA* gene following the mRFP sequence. The plasmid contains a floxed blastocidin resistance cassette. The circular plasmid was electroporated into *Dictyostelium* cells. B and C) Cells carrying the mRFP-AmpA plasmid show a large plaque size characteristic of AmpA overexpressing cells. Scale bars 1000um. D) Development of mRFP-AmpA cells arrests at the mound stage like AmpA overexpressing cells. mRFP-AmpA cells at 20 and 24 hours of development. Very few structures have progressed beyond mound stage at 24 hours. Compare to Wt in Additional File 5 panel D where all cells are culminants by 24 hours. E) Cells carrying the mRFP-AmpA plasmid make a 55Kd protein (mRFP=25Kd and AmpA=30Kd). Arrow indicates the position of the fusion protein. The faint band directly above is fusion protein where the hydrophobic leader had not been efficiently cleaved. The lane containing the Wt cells shows no bands that cross react with the Anti-RFP antibody.
