# Supplementary material for: The adhesion modulation protein, AmpA localizes to an endocytic compartment and influences substrate adhesion, actin polymerization and endocytosis in vegetative Dictyostelium cells
Source: BMC Cell Biol. 2012 Nov 5;13:29. doi: 10.1186/1471-2121-13-29 (PMC3586950; doi:10.1186/1471-2121-13-29)
Supplement: Additional file 13 — AmpA Tap tag fusion proteins show the same distribution of AmpA protein in vesicles throughout the cells and in a perinuclear compartment as the mRFP-AmpA tagged construct. AmpA-tap tagged OE and AmpA-tap tagged Wt strains show a similar distribution of AmpA protein within the cell. Supplemental figure and legend. [file 1471-2121-13-29-S13.pdf]

A

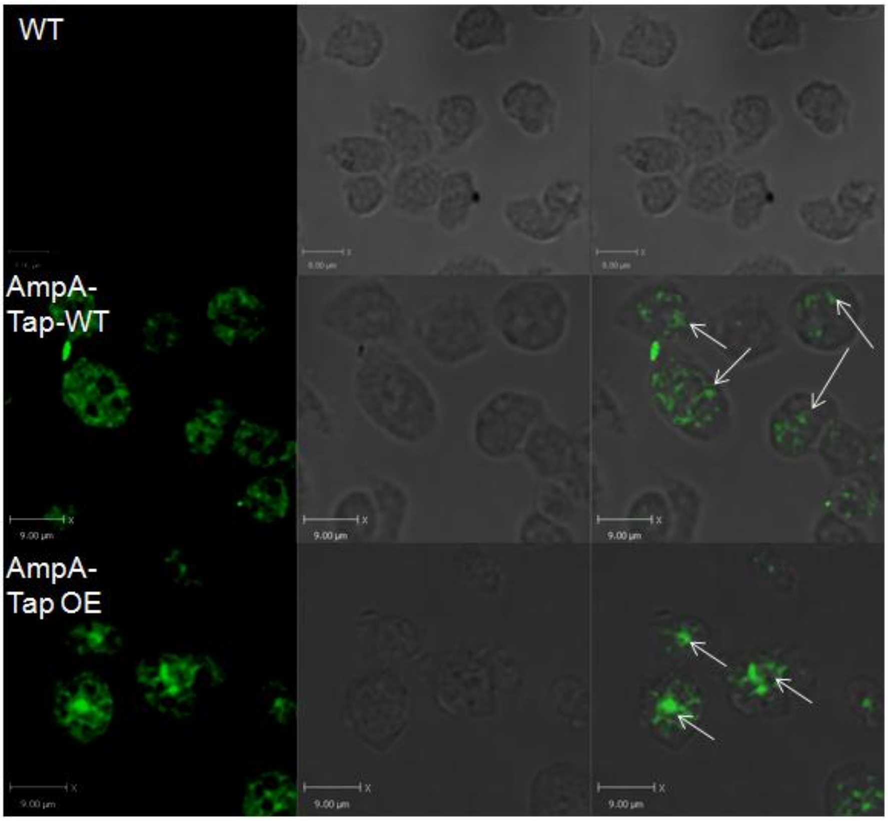

B

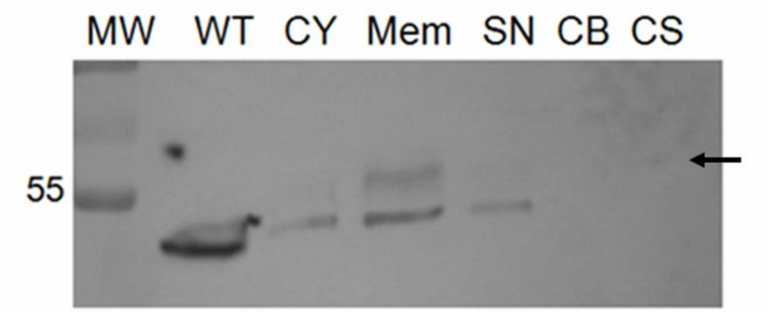

Additional File 13 AmpA Tap tag fusion proteins show the same distribution of AmpA protein in vesicles throughout the cells and in a perinuclear compartment as the mRFP-AmpA tagged construct. AmpA-tap tagged OE and AmpA-tap tagged Wt strains show a similar distribution of AmpA protein within the cell. **A.** Cells were fixed with formaldehyde and permeabilized with methanol. Immunofluorescence images: Green, AmpA-TAP. Primary antibody was rabbit anti-tap, secondary antibody was goat anti rabbit conjugated to Alexa-Fluor 488 (Invitrogen) Arrows indicate areas of AmpA-TAP perinuclear localization. Images in are 3D reconstructions from a Z series of optical sections. Scale bars are 9um **B.** Western blots of fractionations of AmpA-TAP Wt. WT-wild type. CY-Cytoplasm. Mem-Membrane. SN-Soluble Nuclear. CB-Chromatin Bound. CS-Cytoskeletal. The arrow in B indicates the AmpA-tap band. Latter batches of anti-Tap tag antibodies cross reacted with a slightly lower molecular weight band. All of the immunofluorescent images were done with earlier batches of anti-Tap tag antibodies that did not contain this cross reacting activity. It was because of this cross reacting activity that all later immunofluorescence work was done using the mRFP-AmpA fusion protein construct.
