# Supplementary material for: The adhesion modulation protein, AmpA localizes to an endocytic compartment and influences substrate adhesion, actin polymerization and endocytosis in vegetative Dictyostelium cells
Source: BMC Cell Biol. 2012 Nov 5;13:29. doi: 10.1186/1471-2121-13-29 (PMC3586950; doi:10.1186/1471-2121-13-29)
Supplement: Additional file 14 — Overexpression of ampA causes multiple endocytic cups to form repeatedly at the same site. Supplemental figure and legend. [file 1471-2121-13-29-S14.pdf]

A

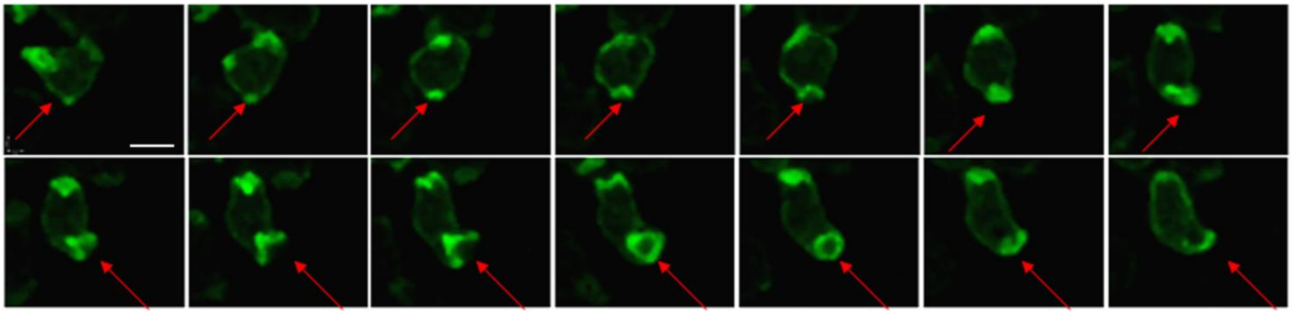

B

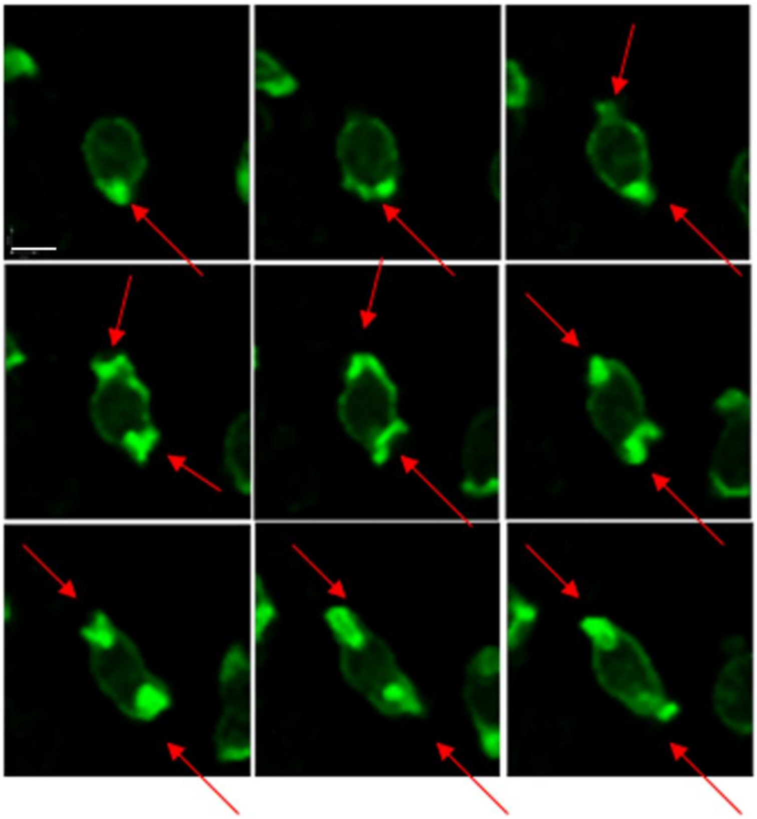

Additional File 14 Overexpression of AmpA causes multiple endocytic cups to form repeatedly at the same site. **A)** And **B)** Two additional OE cells containing the blasticidin resistant ABD-GFP plasmid were placed in chambered cover slips overnight. Images were taken every 20 seconds for 5 minutes. Images are representative optical sections from time courses. Calibration bar = 10um. Arrows indicate sites of formation of multiple successive endocytic cups.
