# Supplementary material for: The adhesion modulation protein, AmpA localizes to an endocytic compartment and influences substrate adhesion, actin polymerization and endocytosis in vegetative Dictyostelium cells
Source: BMC Cell Biol. 2012 Nov 5;13:29. doi: 10.1186/1471-2121-13-29 (PMC3586950; doi:10.1186/1471-2121-13-29)
Supplement: Additional file 18 — Endosomes acidify properly in ampA mutants. Supplemental figure and legend. [file 1471-2121-13-29-S18.pdf]

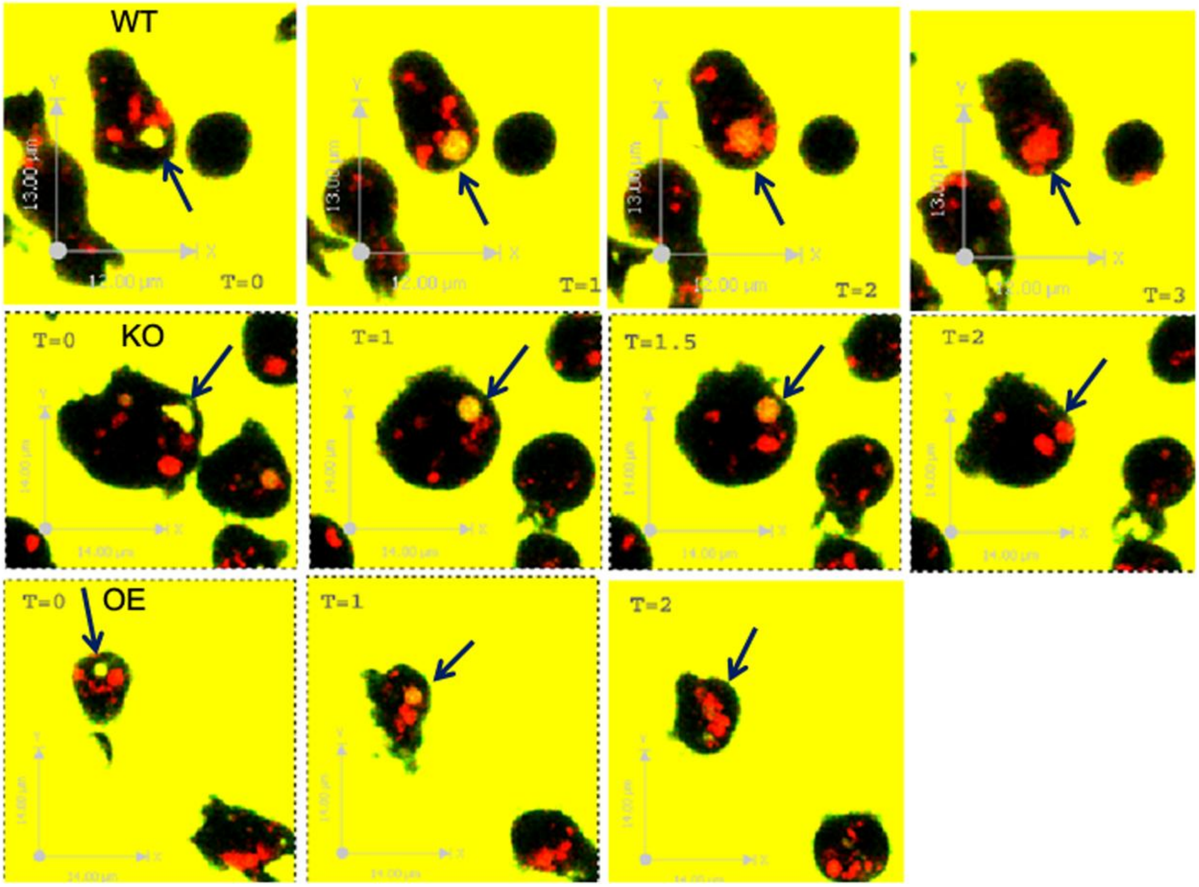

Additional File 18: <u>The endosomes acidify properly in the AmpA mutants</u>
Cells were incubated in chambered coverslips with FITC and TRITC dextran added at 2mg/ml. Images were taken every 15 seconds for 5 minutes. The arrows identify the endosomes. The yellow color indicates that both TRITC and FITC are present and the endosome has not become acidified. As time progresses, the endosomes appear red, indicating that the FITC has been quenched by acidification of the endosome. Images were chosen from the time course to follow one representatvie endosome from neutral to acidified, therefore the time course only shows progression and the numbers are not indicative of speed of acidification. Images are single optical sections from a time course.
