# Supplementary material for: The adhesion modulation protein, AmpA localizes to an endocytic compartment and influences substrate adhesion, actin polymerization and endocytosis in vegetative Dictyostelium cells
Source: BMC Cell Biol. 2012 Nov 5;13:29. doi: 10.1186/1471-2121-13-29 (PMC3586950; doi:10.1186/1471-2121-13-29)
Supplement: Additional file 19 — There is no defect in the formation of contractile vacuoles in ampA mutants. Supplemental figure and legend. [file 1471-2121-13-29-S19.pdf]

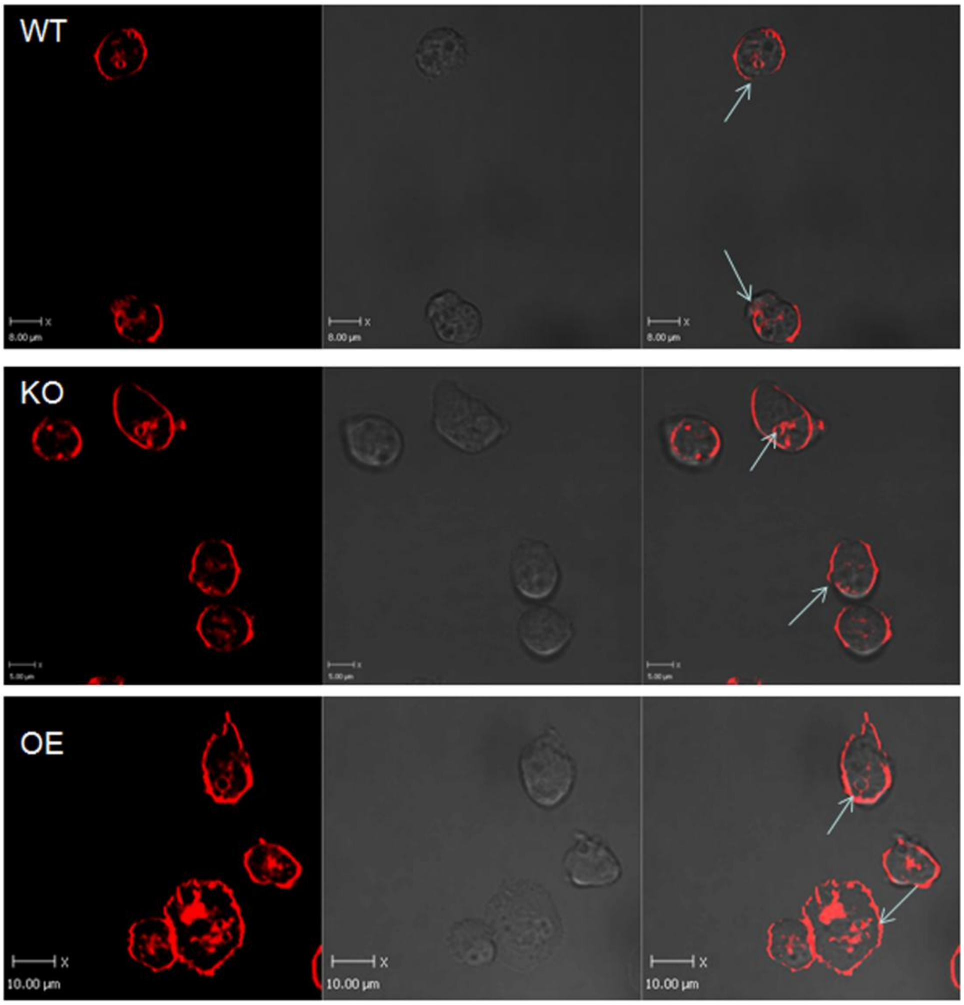

Additional File 19: <u>Contractile vacuoles in AmpA mutants are normal</u>. Cells were incubated in phosphate buffer for 30 minutes prior to addition of FM-64 to a final concentration of 1ug/ml. After a 10 minute incubation period, the cells were imaged. Images represent a single optical section. Scale bars are 10um.
